# Network analysis identifies protein clusters of functional importance in juvenile idiopathic arthritis

**DOI:** 10.1186/ar4559

**Published:** 2014-05-08

**Authors:** Adam Stevens, Stefan Meyer, Daniel Hanson, Peter Clayton, Rachelle Donn

**Affiliations:** 1 Manchester Academic Health Sciences Centre, Royal Manchester Children’s Hospital, Manchester M13 9WL, UK; 2 Stem Cell and Leukaemia Proteomics Laboratory, School of Cancer and Imaging Sciences, University of Manchester, Manchester, UK; 3 Centre for Musculoskeletal Research, University of Manchester, Manchester M13 9PT, UK

## Abstract

**Introduction:**

Our objective was to utilise network analysis to identify protein clusters of greatest potential functional relevance in the pathogenesis of oligoarticular and rheumatoid factor negative (RF-ve) polyarticular juvenile idiopathic arthritis (JIA).

**Methods:**

JIA genetic association data were used to build an interactome network model in BioGRID 3.2.99. The top 10% of this protein:protein JIA Interactome was used to generate a minimal essential network (MEN). Reactome FI Cytoscape 2.83 Plugin and the Disease Association Protein-Protein Link Evaluator (Dapple) algorithm were used to assess the functionality of the biological pathways within the MEN and to statistically rank the proteins. JIA gene expression data were integrated with the MEN and clusters of functionally important proteins derived using MCODE.

**Results:**

A JIA interactome of 2,479 proteins was built from 348 JIA associated genes. The MEN, representing the most functionally related components of the network, comprised of seven clusters, with distinct functional characteristics. Four gene expression datasets from peripheral blood mononuclear cells (PBMC), neutrophils and synovial fluid monocytes, were mapped onto the MEN and a list of genes enriched for functional significance identified. This analysis revealed the genes of greatest potential functional importance to be *PTPN2* and *STAT1* for oligoarticular JIA and *KSR1* for RF-ve polyarticular JIA. Clusters of 23 and 14 related proteins were derived for oligoarticular and RF-ve polyarticular JIA respectively.

**Conclusions:**

This first report of the application of network biology to JIA, integrating genetic association findings and gene expression data, has prioritised protein clusters for functional validation and identified new pathways for targeted pharmacological intervention.

## Introduction

Juvenile idiopathic arthritis (JIA) is a common chronic disease of childhood. It is not a single disease, but a term that encompasses all forms of arthritis, of unknown origin, that begin before the age of 16 years and persist for longer than 6 weeks. It is the commonest form of chronic arthritis in children and the cause of substantial morbidity. JIA is a complex disease with both environmental factors, currently unknown, and genetic contributions to its aetiopathogenesis. Of the clinical subtypes defined by the International League Against Rheumatism (ILAR) classification of JIA [1] oligoarticular (both persistent and extended forms) and rheumatoid factor (RF)-negative (RF-ve) polyarticular arthritis are the predominant forms, accounting for approximately half of all presenting cases in the UK [2]. Factors that influence the susceptibility to, and progression of, these major phenotypic forms are currently unknown. Similarly, treatment options remain limited with steroid joint injections and methotrexate being most frequently employed. However, the relapse rate is often high and over 40% of individuals continue to have active disease as adults [3,4].

Replicated genetic loci from JIA candidate gene, genome-wide association studies (GWAS) and fine mapping genotyping are emerging [5-7]. In addition, a limited number of gene expression studies have attempted to establish changes in gene expression patterns with oligoarticular or with RF-ve polyarticular JIA subtypes [8-10]. Single nucleotide polymorphism (SNP) association studies, be it by a candidate gene or a GWAS approach, can identify variations that are associated with a particular complex trait. However, multiple constraints exist in extrapolating these observations forwards to any biological significance. SNP analysis, in the main, is done only at the single locus level. The associated variant may not be the functional polymorphism. Furthermore, SNPs associated with complex traits such as JIA, typically have small effect sizes and account for only a very small fraction of the genetic risk [11]. Thus, for most complex diseases, including JIA, an understanding of the underlying biology, leading to better diagnosis and treatment, is unlikely to arise by detailing the functional consequence of individual SNPs. Therefore, a major challenge for JIA, as for other complex diseases, is the integration of high-throughput omic datasets and subsequent identification and prioritisation of disease-associated loci for additional investigation.

JIA, most likely, arises as a result of abnormalities in genes, but more specifically, via the manifestation of perturbations in multiple protein networks that integrate cellular processes, and those that also link cells within tissues, and tissues within organ systems. An innovative approach to identifying key contributing genetic loci, and potential mechanisms of disease in patients with JIA, is the use of network biology. Network analysis (see Table 1) can allow the summation of various interactions and interdependencies between SNP associations and gene expression data [12]. Defining such a network structure is relevant for biological function. Topologically derived networks have interacting proteins that tend to be co-evolving [13], co-functional [14], and co-expressed [15,16].

**Table 1:** Glossary

**Term**	**Definition**^**a**^
Bottleneck	A network position that limits the performance of the system. Described by the mathematical idea of *betweeness centrality* - a description of the number of paths travelling through a node in a network. An example would be a rate-limiting enzymatic reaction.
Cluster	A measure of the tendency of network nodes to form groups. It manifests from a high density of edges between nodes. In biological networks a cluster could represent a protein complex.
Connectivity/degree	The number of connections (see edge) made by any node within a network.
Edge	An interaction between two nodes. Commonly represents a protein:protein interaction but can also be representative of other biological phenomena such as co-expression.
Interactome	A network representing a whole set of direct and/or indirect interactions related to a specific biological phenomenon.
Minimal Essential Network (MEN)	Top 10% of network protein nodes, as scored by connectivity and bottleneck network properties. Corresponds to highly functionally related positions within the network.
Network analysis	Analysis that relates the structural and mathematical properties of a network to its function.
Node	A vertex within a network. In biological networks a node will usually be a gene, protein or metabolite.
Seed genes	A starting set of genes or metabolites of interest that are used to generate an interactome model by inferring joining interactions algorithmically.

^**a**^Further technical references are given in Stevens *et al.*, [33].

It is recognised that for the majority of SNPs with a potential functional role in disease the genome-wide significance threshold for disease association (*P* <1 × 10^−8^) may not be reached [17]. Therefore, despite not individually reaching genome-wide statistical significance, SNPs that cluster in networks can inform the underlying biology of a complex genetic disease such as JIA. Studies now describe how network biology place signature changes within the human interactome, uncovering some of the complexities of human diseases [12,18,19]. Furthermore, network analysis can inform drug discovery and drug targeting for complex genetic diseases such as JIA [20,21].

In this present study we have performed network analysis focussed on oligoarticular and RF-ve polyarticular arthritis, integrating genetic association data with gene expression data. This has been used to derive a JIA interactome, and clusters of proteins of functional relevance.

## Methods

**Figure 1:**
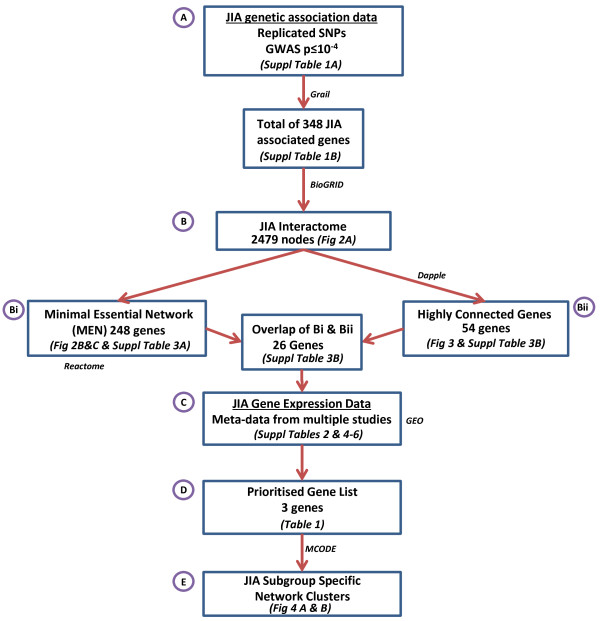
**Overview of the workflow used to identify protein clusters of functional importance in rheumatoid factor-negative (RF-ve) polyarticular and oligoarticular juvenile idiopathic arthritis (JIA).** This figure outlines each of the steps taken and gives cross references to the specific tables and figures that provide further details of that particular step in the process. **(A)** JIA genetic association data of replicated single nucleotide polymorphisms (SNPs) associated with RF-ve polyarticular JIA or oligoarticular JIA and JIA genome-wide association data findings were ascertained. SNPs were mapped to genes using Gene Relationships Across Implicated Loci (GRAIL) software (n = 348) (Additional file 1: Table S1A and B). **(B)** A JIA interactome network model (JIA Interactome) was inferred from JIA-associated genes using the BioGRID database (Figure 2A). **(Bi)** The top 10% of nodes, ranked by connectivity and bottlenecks, were used to derive a minimal essential network (MEN) of 248 genes (Figure 2B and Additional file 3: Table S3A). The Reactome database and spectral partition clustering was used to determine clusters of genes within the MEN (Figure 2C). **(Bii)** The Disease Association Protein-Protein Link Evaluator (Dapple) algorithm was used to identify Highly Connected Genes (Figure 3 and Additional file 3: Table S3B). The overlap of genes between **Bi** and **Bii** was determined (n = 26 genes) (Additional file 3: Table S3B). **(C)** JIA gene expression data from published sources, collated from the Gene Expression Omnibus database (GEO) (Additional file 2: Table S2 and Additional file 4: Table S4, Additional file 5: Table S5, Additional file 6: Table S6), was integrated with the overlap of the genes from **Bi** and **Bii**. **(D)** A prioritised list of JIA-associated genes with functionally associated network properties was identified from the integration of JIA genetic association and gene expression data (n = 3 genes) (Table 2). **(E)** MCODE was used to determine RF-ve polyarticular and oligoarticular JI-specific network clusters, protein:protein interactions of functional relevance (Figure 4A and B). GWAS, genome-wide association studies.

An overview of the approach taken is shown in Figure 1, and the sections below are labelled in correspondence with this figure. These sections detail the key processes and stages in the work flow for the formulation of the JIA subgroup specific network clusters of functional importance.

### Genetic association data

Initially, PubMed [22] and Web of Science [23] searches were conducted and only validated SNP associations from studies of oligoarticular and RF-ve polyarticular JIA were selected. These included multiple well-replicated loci initially associated with adult rheumatoid arthritis found to be significantly associated with JIA [24-30]. In addition, the top findings (*P* ≤1 × 10^−4^) from the largest GWAS study in JIA have been used [6], together with loci replicated by dense genotyping of immune-related disease regions in JIA [7]. SNPs were assigned to genes using an algorithm within Gene Relationships Across Implicated Loci (GRAIL) [31,32] (Additional file 1: Table S1A). This identified a total of 348 JIA-associated genes (Figure 1A). These 348 JIA-associated genes were the seed genes, that is, known genes that are used to generate an interactome model from all known protein-protein interactions (Additional file 1: Table S1B).

### Building the JIA interactome

A JIA interactome was derived from the genetic association data using two independent methods (Figure 1Bi and Bii). Interactome modelling uses seed genes, in this case JIA-associated genes, to infer a network of protein:protein interactions, which are derived from known interactions between the seed genes and their near neighbours [33].

Bi: the first approach used the BioGRID Cytoscape Plugin [34] to create a filter for the BioGRID human interactome model (3.2.99), based on the JIA-associated genes. The resultant JIA interactome was visualised in Cytoscape (version 2.8.3). The Cytohubba Cytoscape Plugin was used to provide topological analysis of this network and connectivity and bottlenecks were calculated. The JIA interactome was then ranked for both connectivity and bottleneck properties and these were used to evaluate the relative importance of each node [35,36], and to generate a minimal essential network (MEN) [33,37,38]. The top 10% of network nodes was chosen to define the MEN (Figure 1Bi), as this represents the most functional elements of the network [33,37]. The Reactome FI Cytoscape 2.8.3 Plugin [39,40] was then used to determine clusters - distinct groups of protein:protein interactions [41] and to analyse the functional enrichment of biological pathways within the MEN.

Bii: the second approach utilised the Disease Association Protein-Protein Link Evaluator (Dapple) algorithm [42,43] and InWeb database [44]. This approach revealed highly connected genes within the JIA interactome by testing the significance of biological networks using a permutation method (10,000 permutations). The overlap of genes from Bi and Bii was determined using a Venn diagram (Partek Genomics Suite).

### Gene expression data was integrated with the JIA interactome

Gene expression analysis was conducted on a library of gene expression datasets from children with JIA collated from the NCBI Gene Expression Omnibus (GEO) database (Additional file 2: Table S2). Selection was made based on the phenotypic criteria of oligoarticular or RF-ve polyarticular JIA and on control data being accessible.

Gene expression studies were downloaded from GEO [45] and annotation was assessed using QlucoreOmics Explorer 2.2 (Lund, Sweden). Sample comparisons were grouped for disease versus controls, or disease versus disease, using the data in the original peer-reviewed study. Available covariates (confounding factors) as provided in the published data were used. Four separate gene expression datasets were utilised [GEO:GDS711 [9], GSE:11083 [10], GSE:20307 [8], GSE:17755] [46]. The full details of these datasets are given in Additional file 2: Table S2.

Probe-to-gene assignment was made using the appropriate Affymetrix annotation file (Netaffx.com). Dimensional scaling using principal components analysis (PCA) and Iso-map multidimensional scaling [47,48] were used to demonstrate data homogeneity (Qlucore Omics Explorer 2.2) and to identify outliers using cross-validation. Analysis of variance (ANOVA) was used to determine differential gene expression between groups (*P* ≤0.05) and functional associations were cross-referenced using Ingenuity Pathway Analysis software (IPA). Overlap of gene expression data sets was performed separately for both up- and down-regulated genes using Venn diagrams (Partek).

### Deriving a list of prioritised genes of functional relevance

Genes identified within the MEN by network topology (Figure 1Bi) were overlapped with highly connected genes defined using Dapple (*P* ≤0.05) (Figure 1Bii). This step identified network positions with putative functional importance using two semi-independent analytical approaches. This overlap was then combined with gene expression data (Figure 1C) to derive a prioritised list of genes of functional relevance.

### Identification of JIA subgroup-specific network clusters

RF-ve polyarticular JIA and oligoarticular JIA clusters were identified from the JIA interactome using the MCODE algorithm plugin for Cytoscape [49]. These clusters identify the most highly related RF-ve polyarticular and oligoarticular JIA subgroup-specific genetic and transcriptomic data within the JIA interactome.

### Statistics

Assessment of pathway associations was performed by the hypergeometric test using the Benjamini-Hochberg false discovery rate (FDR) correction (FDR correction-modified *P*-value ≤0.05) [50].

## Results

### Deriving a minimal essential network of biologically relevant JIA genes

**Figure 2:**
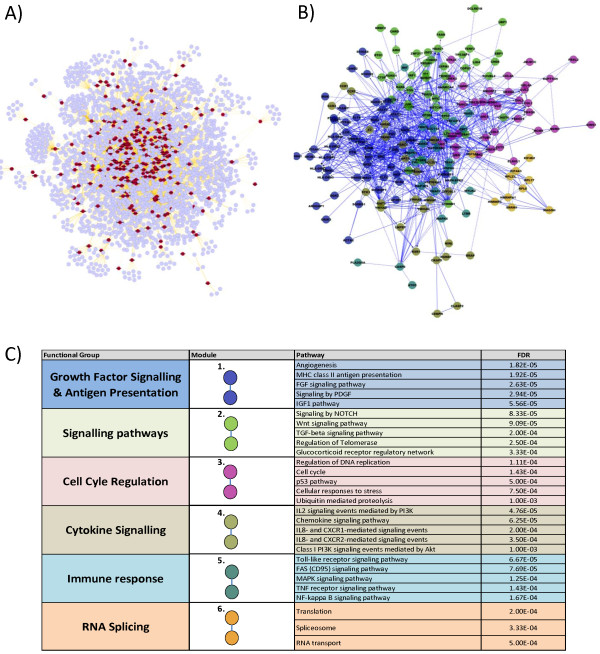
**Network analysis of juvenile idiopathic arthritis (JIA)-associated genes. (A)** A collated list of 348 JIA-associated genes was used to derive an interaction network inferred from the BioGRID model of the human interactome (3.2.99); red = JIA-associated gene, blue = inferred interaction (Figure 1B). **(B)** Minimal essential network of top 10% of the genes from the JIA interactome, ranked by connectivity and bottleneck, colours represent clusters of related genes calculated by spectral partition clustering [41] (Figure 1Bi). **(C)** Biological pathways associated with minimal essential network (MEN) clusters (colour of cluster relates to Figure 2B), false discovery rate (FDR) *P*-value of hypergeometric test (*P* ≤0.05) (Figure 1Bi). *MHC* Major histocompatibility complex, *FGF* Fibroblast growth factor, *PDGF* Platelet-derived growth factor *IGF-1* Insulin-like growth factor 1, *TGF* beta Transforming growth factor beta, *IL-2* Interleukin 2, *IL-8* Interleukin 8 *PI3K* Phosphoinositide 3-kinase, *TNF* Tumour necrosis factor, *CXCR1* chemokine (C-X-C motif) receptor 1, *CXCR2* chemokine (C-X-C motif) receptor 2.

A collated list of 348 JIA-associated seed genes was used to derive a JIA interactome inferred from the BioGRID human interactome database (3.2.99). This consisted of the 348 seed genes (marked red in Figure 2A) along with their immediate inferred interaction partners (protein:protein interactions - purple in Figure 2A). This gave a network of 2,479 proteins and 4,147 edges (Figure 2A). The top 10% of nodes, ranked by connectivity and bottleneck properties were selected to generate a minimal essential network (MEN) (Figure 2B, Additional file 3: Table S3A). The MEN represents the most functionally related components of a network [33,37].

The JIA-associated clusters (same colour code in Figure 2B and C; listed in Additional file 3: Table S3A) within the MEN were mapped onto biological pathways using the Reactome Cytoscape plugin to ascertain function. Six clusters were identified within the MEN, which had clear associated biological pathways (FDR, *p* ≤0.05). These include pathways related to growth factor signalling and antigen presentation, regulation of the cell cycle and cytokine signalling (Figure 2C).

### Permutation analysis of network topology

**Figure 3:**
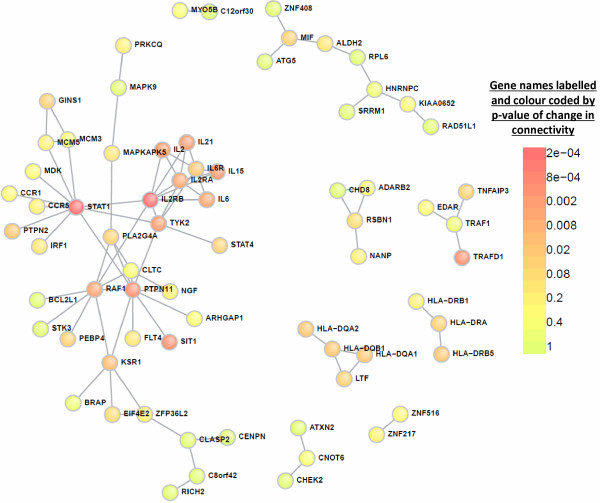
**Changes in connectivity of juvenile idiopathic arthritis (JIA)-associated genes.** The Disease Association Protein-Protein Link Evaluator (Dapple) algorithm was used to generate an inferred interactome network from the 348 JIA-associated genes, an iterative process was then used to generate random networks and significant changes in seed gene connectivity were calculated. The JIA-associated seed genes are shown coloured by significance of deviation of observed network connectivity from expected (red = highly significant to green = not significant) (Figure 1Bii).

Network analysis of the JIA-associated genes was also performed using the Dapple algorithm [42]. This was done to calculate changes in connectivity in the inferred network greater than those expected by chance. First an inferred interaction network was derived using the JIA genetic associations as seed genes (Figure 1B). Permutation analysis of this inferred network demonstrated an increase of seed gene connectivity (*P* <0.002). The seed genes were ranked, by *P*-value of increased connectivity (colour scale of ranking, Figure 3; all seed genes listed in Additional file 3: Table S3B).

### Prioritisation of JIA-associated genes by integration of the JIA interactome with gene expression data

The overlap between the genes present within the MEN and the highly connected loci, identified by Dapple (Figure 1Bi and Bii) was established. This identified 26 genes of the 248 genes that occur in the MEN, that are ranked by Dapple with a *P*-value ≤0.05 (coloured orange in Additional file 3: Table S3B). These 26 genes represent the most highly connected, and therefore, potentially most functionally important loci, derived from the genetic association findings. Then, in order to prioritise these JIA-associated genes for future functional investigation, gene expression data were aligned (Figure 1C, Figure 2A - red nodes). Four different gene expression series were identified that provided data from RF-ve polyarticular, oligoarticular JIA and controls in previously published studies (GEO). These datasets allowed comparison between gene expression in peripheral blood mononuclear cells (PBMCs), neutrophils or synovial fluid monocytes (SFM) (Additional file 2: Table S2).

#### RF-ve polyarticular JIA versus controls

RF-ve polyarticular JIA gene expression data were compared with controls. Four comparisons were made using PBMC data and one using neutrophils (Additional file 2: Table S2). Comparisons were made separately between up-regulated and down-regulated genes. To increase confidence in the statistical interpretation of the data, and to reduce confounding effects, only genes with significant differential expression in at least three out of the five of these comparisons (Additional file 4: Table S4) were mapped onto JIA-associated genes ranked by network properties. In total this comprised 325 genes; 138 up-regulated and 187 down-regulated (Additional file 4: Table S4).

#### Oligoarticular JIA versus controls

Oligoarticular JIA gene expression was compared with controls using PBMC expression data (Additional file 2: Table S2). Comparisons were made separately between up-regulated and down-regulated genes. Genes with significant differential expression in both comparisons (Additional file 5: Table S5) were then mapped onto the list of JIA-associated genes ranked by network properties. There were a total of 150 genes with altered gene expression, 46 being up-regulated and 104 down-regulated (Additional file 5: Table S5).

#### Oligoarticular JIA versus RF-ve polyarticular JIA

Oligoarticular was compared to RF-ve polyarticular JIA gene expression data. Two PBMC and one synovial fluid mononuclear cell expression data set were utilised (Additional file 2: Table S2). Comparisons were made separately between up-regulated and down-regulated genes. Genes with significant differential expression in all three comparisons (Additional file 6: Table S6) were then mapped onto the list of JIA associated genes ranked by network properties. A total of 108 genes were changed, 88 were up-regulated and 20 were down-regulated (Additional file 6: Table S6).

### JIA subgroup-specific loci of greatest functional relevance

The JIA subgroup specific loci of greatest functional relevance were identified as those loci that were present within the overlap of the MEN and highly connected genes (Figure 1Bi and Bii) with concomitant variation in gene expression (Figure 1C). These loci are listed in Table 2.

**Table 2:** Genes of greatest potential functional relevance determined by network analysis

	**JIA-associated genes with correlated expression data that lie within the minimal essential network (MEN)**	**Dapple *P*-value**
RF-ve polyarticular JIA compared with controls	*KSR1*	0.0088
Oligoarticular JIA compared with controls	*PTPN2*	0.0112
Oligoarticular compared with RF-ve polyarticular JIA	*STAT1*	0.0002

Genes present within the MEN which also show altered gene expression in either rheumatoid-negative (RF-ve) polyarticular juvenile idiopathic arthritis (JIA) compared with controls, oligoarticular JIA compared with controls, or between oligoarticular compared with RF-ve polyarticular JIA are listed. Presence in the MEN indicates functional priority and the Disease association protein-protein link evaluator (Dapple) *P*-value assesses the statistical significance of network connectivity compared to what would be expected by chance.

The network analysis identified *KSR1* as the locus of greatest potential functional relevance in RF-ve polyarticular JIA, and *PTPN2* for oligoarticular JIA (Table 2). *Signal transducer and activator of transcription 1* (*STAT1*) is differentially involved in oligoarticular versus the polyarticular subgroup. It has increased connectivity, indicating a primary functional role, in oligoarticular JIA compared with RF-ve polyarticular disease (Table 2).

### Prioritised genes form JIA subgroup-specific functional clusters

In order to determine functional clusters, that is, distinct groups of protein:protein interactions in relation to the key prioritised genes, KSR1 for RF-ve polyarticular JIA and Protein tyrosine phosphatase, non-receptor type 2 (PTPN2) for oligoarticular JIA the MCODE algorithm was applied to the JIA interactome (Figure 1E). The MCODE algorithm identifies highly connected regions of biological networks representing clusters of related function that include protein:protein interaction complexes [49]. This approach identified two high scoring clusters each containing <25 proteins. High-scoring clusters have a high density of local connections and thus they represent a network region with related function [33,51,52]. The cluster score generated by an algorithm such as the MCODE algorithm used in this case, ranks network clusters as a way of prioritising function.

**Figure 4:**
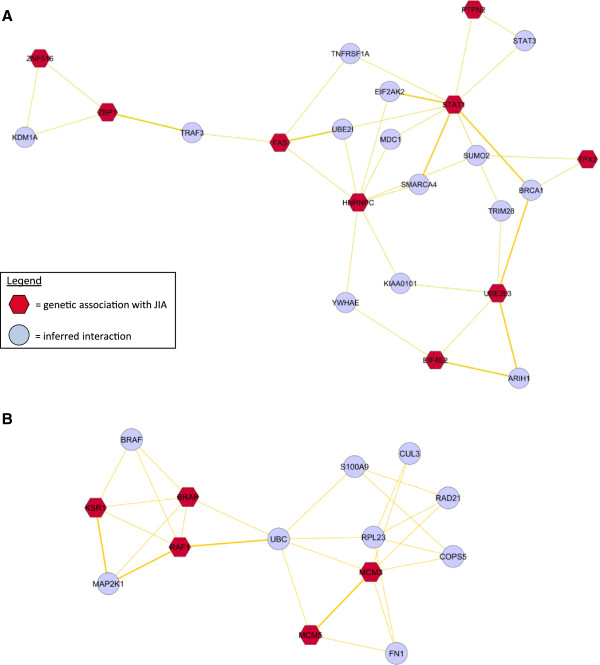
**Identification of clusters of highly connected nodes within the juvenile idiopathic arthritis (JIA) interactome.** Two clusters were identified using the MCODE clustering algorithm. **(A)** The oligoarticular JIA cluster includes PTPN2 and STAT1; **(B)** the RF-ve polyarticular related cluster contains KSR1. JIA-associated genes = red hexagons, blue circles = inferred genes within the JIA interactome (Figure 1E).

A cluster of 23 proteins contained PTPN2 and STAT1 (Figure 4A). Both *PTPN2* and *STAT1* are JIA-associated genes (Additional file 1: Table S1) with concomitant significant changes in gene expression in oligoarticular JIA patients. *PTPN2* was significantly down-regulated in oligoarticular JIA compared with controls; *STAT1* significantly increased in expression in oligoarticular compared to RF-ve polyarticular JIA cases (Additional file 6: Table S6). The main biological pathways associated with this cluster are cytokine signalling (*P* <1.8 × 10^-4^) and DNA repair (*P* <1.4 × 10^-3^).

A second functional cluster of 14 proteins was identified that contained KSR1 (Figure 4B). *KSR1* is a JIA-associated gene (Additional file 1: Table S1) with significant increased expression in RF-ve polyarticular JIA patients (Additional file 4: Table S4). The main biological pathways found to be associated with the KSR1-related cluster are the RAF/MAP kinase cascade (*P* <8.9 × 10^-5^), cell cycle checkpoints (*P* <5.6 × 10^-4^), DNA replication (*P* <1.3 × 10^-4^) and cytokine signalling (*P* <1.2 × 10^-2^).

## Discussion

Recognising the necessity to move away from an approach focussed around the determination of SNP function *per se* we have utilised network biology approaches to reveal highly connected regions within a derived JIA interactome in order to identify the interplay of molecular elements that leads to the phenotypic expression of RF-ve polyarticular JIA or to oligoarticular JIA.

We have used a comprehensive set of genetic loci as the starting point to build the network. These loci are replicated SNP associations from candidate genes and the top statistical associations from recent GWAS and fine-mapping studies. There are limited collections of JIA patients worldwide to allow for subsequent GWAS or future GWAS meta-analysis. Given the premise that gene-gene interactions contribute to complex diseases, combining modest association signals from GWAS analysis with biological data to build networks can help to detect the joint effects of multiple genes [53]. Also, Hua *et al.* recently described the relevance of incorporating GWAS SNPs with a *P*-value of lower than 0.01 in network analysis [54].

The genetic network was initially built using the BioGRID database. Subsequently, a MEN, representing the core genetic clusters related to the JIA phenotype, was derived using both connectivity and bottleneck network properties [12,33,36-38,55]. Six biological groupings were associated with the MEN, with a predominance of immune-related and cellular signalling pathways being represented (Figure 2). In addition, we utilised the approach described by Rossin *et al.*[42] to provide a statistical measure to the MEN.

The value of network biology is the inter-relational display of multiple omic datasets [56]. An integrated, multi-omic approach reduces noise in the statistical interpretation [57]. Furthermore, network biology focuses on the importance of clusters [58-61] as a measure of similarity rather than simply an overlap of gene sets. It is also an approach that is robust to random variation in the data [62] and to variation over different sizes of networks [60].

Our strategy has been to build the network from genetic association findings and to refine and validate the SNP data by overlaying stringently derived gene expression data. In order to have the most stringent refinement of the network we integrated the gene expression data with the MEN [33,37,38], and then the Dapple algorithm [42] was again employed to rank the gene expression loci that aligned to the MEN, to provide a further level of statistical robustness. This analysis identified JIA subgroup-specific loci.

The key seed gene for RF-ve polyarticular JIA is the scaffold kinase suppressor of Ras (*KSR1*). In three of the five gene expression datasets *KSR1* was over-expressed in RF-ve polyarticular JIA (Additional file 4: Table S4). KSR1 acts as a location-regulated scaffolding protein connecting MEK to RAF. It promotes MEK and RAF phosphorylation and activity through assembly of an activated signalling complex. By itself, however, it has not been demonstrated to have kinase activity. Importantly, Fusello *et al*. established that KSR1 knockout mice have reduced susceptibility to rheumatoid arthritis [63]. As KSR1-deficient T cells are functionally impaired [64] Fusello tested *Ksr*-deficient mice using a passive transfer model of arthritis. Their findings showed that the induction of arthritis is impaired in the absence of KSR1 and that this gene plays a role in ERK activation during inflammatory and stress responses both *in vitro* and *in vivo*. Using the MCODE algorithm, KSR1 interacts with a sub-cluster of 14 other proteins including a functional relationship with RAF1 (Figure 4B). We propose that this sub-cluster is of key functional relevance to the pathogenesis of RF-ve polyarticular JIA.

For oligoarticular JIA *PTPN2* was the seed gene of importance. *PTPN2* was significantly altered (down-regulated) in expression and mapped to the MEN (Additional file 5: Table S5). The cluster in relation to PTPN2 is shown, (Figure 4A), and consists of 23 proteins, the majority of which have a role in cytokine signalling and DNA repair.

*PTPN2* encodes for the ubiquitously expressed T cell protein tyrosine phosphatase (TCPTP), a JAK/STAT and growth factor receptor phosphatase that has been linked with the pathogenesis of type 1 diabetes mellitus, rheumatoid arthritis and Crohn’s disease by GWAS findings of non-coding SNP associations. Mouse and human studies have shown that reduced expression of TCPTP may drive autoimmune pathologies by enhancing signalling downstream of the T cell receptor (TCR), cytokines, or growth factors to produce a pro-inflammatory cytokine milieu [65].

Hinks *et al.* reported the *C5orf56-IRF1* region to show differential association between oligoarticular and RF-ve polyarticular JIA [7]. IRF1 is present in the MEN (Figure 2B & Additional file 3: Table S3A) and *IRF1* gene expression is significantly increased in oligoarticular compared to RF-ve polyarticular JIA (Additional file 6: Table S6). It has borderline significance in post iterative modelling (Dapple) (*P* = 0.07). *STAT1,* a signal transducer and transcription activator that mediates cytokine activity and growth factor, mapped within the MEN, was differentially expressed, with higher expression occurring in oligoarticular compared to RF-ve polyarticular disease, and met the FDR cut off for Dapple assignment of *P* ≤0.05. STAT1 is present in the oligoarticular JIA but not the RF-ve polyarticular JIA sub-cluster. It appears, therefore, to represent a gene that differentiates between the oligoarticular and RF-ve polyarticular clinical subtypes. Further evaluation of IRF1 and STAT1 in persistent versus extended oligoarticular JIA is not possible as neither the genetic association nor the gene expression studies have classified oligoarticular JIA cases in this way.

The main observations from the GWAS analysis by Thompson *et al*. [6] relate to the *C3orf1* and *JMJD1C* genes. We find the proteins for these loci to be peripheral within our interactome network and they are not prioritised by Dapple. This implies that although these loci may have a role in JIA pathogenesis their lack of connectivity limits their druggable potential (reviewed in [66]).

Functional assessment of the RF-ve polyarticular and oligoarticular clusters needs to be determined. Mounting evidence indicates that biological systems are organised as modular networks, in which genes, proteins, metabolites and other factors operate in groups rather than as single entities. There is increasing recognition that transcription factors, micro RNAs, DNA methylation and chromatin remodelling regulate the expression of large numbers of genes in concert [67].

It is intriguing that preliminary analysis using the TargetScan human database [68] shows that the three candidate genes prioritised by this study (*KSR1*, *PTPN2* and *STAT1*) are co-regulated by miR-30a-5p (*P* = 5.3 × 10^-4^). Whilst hundreds of miRNAs have been identified to be dysregulated in various disease tissues, only a fraction have been functionally characterised. mIR 30a-5p been shown to be differentially expressed and to have biological function regulating expression of genes in a number of diseases including systemic lupus erythematosus (SLE) [69], rodent models of diabetes mellitus [70] glioma growth [71] and carcinoma of the colon [72]. Individual miRNAs can regulate several hundered transcripts with effector molecules that function at various sites within cellular pathways and networks, making them master regulators of the genome. miRNA-based therapy is progressing [73]. miRNAs of potential therapeutic value are those that yield satisfactory efficacy in disease model systems and mechanistic data to allow accurate placement of the miRNA into disease-related pathways. The network analysis we have conducted so far allows us to begin to investigate the capacity of mIR 30a-5p in a targeted way in JIA.

Repositioning of drugs is another important therapeutic advantage that can be maximised from network analysis [20,21]. The KSR1 sub-cluster (Figure 4B) could be therapeutically targeted in RF-ve polyarticular JIA patients, using small-molecule inhibitors of RAF1. Repurposing a drug such as sorafenib (nexavar), currently licensed for the treatment of renal and hepatocellular carcinoma, for the management of RF-ve polyarticular JIA could therefore be considered.

There are limitations to the present analysis. First, we have utilised data from a single GWAS, that is, the only one that currently exists for JIA, and included SNP associations of *P* ≤1 × 10^−4^. The widening of input data for the generation of initial interactome networks is, however, acceptable and valid [53,54]. In addition, we have used the data from dense genotyping of immune-related loci [7]. Although only a proportion of the genes potentially involved in JIA pathogenesis may be immune-related, such pathways were highly significant within our network (Figure 2C). Interactome network models can be used to map associated biological function in a sensitive and robust manner via network clusters using stringent downstream analysis [33]. This is the strategy we have applied. Furthermore, if subsequent JIA GWAS or replication of new candidate gene associations do emerge, our network model can readily be adapted to incorporate the additional findings.

Second, there were only four gene expression studies suitable for inclusion. However, these four studies allowed comparisons across different cell populations (PBMCs, neutrophils, synovial fluid monocytes). Including gene expression data, significant across these sites, with the genetic association data and network analysis is a robust approach to identifying major contributing loci to the disease phenotype. Such integrative approaches have improved statistical power [56,57].

Third, the functional significance of the protein clusters remains to be established. The collection of biological material from oligoarticular and RF-ve polyarticular JIA patients is now underway to facilitate this.

## Conclusions

To our knowledge this is the first use of network analysis to integrate JIA genetic and expression data for the prioritisation of loci for further functional assessment. The value of the use of network biology is to increase confidence in the observations of differentially expressed genes, and genetic findings, by correlation with functionally related network structure. This enhances the identification of functional units or clusters and, critically, can inform the targeting of new therapies. We have used different software-based methods (BioGRID, Ingenuity, Reactome) to infer relationships of differentially expressed genes and genetic association data with known interactions in the literature or protein databases. Dapple has been used at two stages in the analysis to add statistical robustness to our findings.

Deciphering the basis of complex pathologies such as JIA is challenging and although certain progress has been made, much remains to be understood. Deriving a JIA interactome model and sub-clusters, as described herein, offers the potential for a new era in addressing the mechanisms and future interventions for this chronic, disabling condition.

## Abbreviations

ANOVA: analysis of variance; C3orf1: Chromosome 3 open reading frame 1; C5orf56: Chromosome 5 open reading frame 56; Dapple: Disease Association Protein-Protein Link Evaluator; FDR: false discovery rate; GEO: Gene Expression Omnibus; Grail: Gene Relationships Across Implicated Loci; GWAS: genome-wide association studies; ILAR: International League Against Rheumatism; IPA: Ingenuity pathway analysis; IRF1: Interferon regulatory factor 1; JIA: juvenile idiopathic arthritis; JMJD1C: Jumonji domain containing 1C; KSR1: Kinase suppressor of Ras; MEN: minimal essential network; PBMC: peripheral blood mononuclear cell; PCA: principal components analysis; PTPN2: Protein tyrosine phosphatase, non-receptor type 2; RF: rheumatoid factor; SFM: synovial fluid monocytes; SNP: single nucleotide polymorphism; STAT1: Signal transducer and activator of transcription 1.

## Competing interests

The authors declare that they have no competing interests.

## Authors’ contributions

AS and RD conceived the idea for integrating genetic and transcriptomic datasets to investigate JIA. AS performed the analyses and developed the minimal essential network as a study tool. DH contributed to and supported network analysis of data. AS, RD, SM and PC designed the analysis plan and the presentation of the data. AS and RD led the writing of the manuscript with input from all authors. All authors read and approved the final manuscript.

## Supplementary Material

Additional file 1: Table S1 Single nucleotide polymorphism (SNP) datasets: replicated loci showing association with oligoarticular and rheumatoid factor-negative (RF-ve) polyarticular juvenile idiopathic arthritis (JIA) were identified from published literature and collated with top genome-wide association studies (GWAS) findings **(A)**. The collated list of genes **(B)** was used as the seed genes for the network analysis and the generation of the JIA interactome (Figure 1). Click here for file

Additional file 2: Table S2 Gene expression data: juvenile idiopathic arthritis (JIA) gene expression datasets were identified from the Gene Expression Omnibus (GEO). Details of the datasets include sample and control numbers, comparisons made, number of probe sets identified by analysis of variance (ANOVA) as significant (*P* <0.05), number of individual genes with a gene expression change, confounding factors included as covariants in the ANOVA and type of Affymetrix Chip. PBMC, peripheral blood mononuclear cells: SFM, synovial fluid mononuclear cells; Neut, neutrophils. Overlap groups used for comparisons are colour coded. Click here for file

Additional file 3: Table S3 Network topology of the juvenile idiopathic arthritis (JIA) interactome: **(A)** Genes from the minimal essential network (MEN), that is, the top 10% of genes from the JIA interactome (ranked by degree (Deg) and bottleneck score (BN)). **(B)** JIA seed genes from the JIA interactome ranked by significance of deviation of observed network connectivity from expected (Disease Association Protein-Protein Link Evaluator (Dapple) algorithm, *P* ≤0.05). Orange = also present in minimal essential network (Figure 2B). Click here for file

Additional file 4: Table S4 Rheumatoid factor-negative (RF-ve) polyarticular juvenile idiopathic arthritis (JIA) versus control gene expression: overlap of genes in RF-ve polyarticular JIA compared to controls present in three of the five gene expression datasets analysed. Datasets described in Additional file 2: Table S2. Red = up-regulated in JIA, green = down-regulated in JIA. Click here for file

Additional file 5: Table S5 Oligoarticular versus control gene expression: overlap of genes in oligoarticular juvenile idiopathic arthritis (JIA) compared to controls present in both gene expression datasets analysed. Datasets described in Additional file 2: Table S2. Red = up-regulated in JIA, green = down-regulated in JIA. Click here for file

Additional file 6: Table S6 Oligoarticular versus rheumatoid factor-negative (RF-ve) polyarticular juvenile idiopathic arthritis (JIA) gene expression: overlap of genes in oligoarticular JIA compared to RF-ve polyarticular JIA present in both gene expression datasets analysed. Datasets described in Additional file 2: Table S2. Red = up-regulated in JIA, green = down-regulated in JIA. Click here for file
