# Supplementary material for: Network analysis identifies protein clusters of functional importance in juvenile idiopathic arthritis
Source: Arthritis Res Ther. 2014 May 8;16(3):R109. doi: 10.1186/ar4559 (PMC4062926; doi:10.1186/ar4559)
Supplement: Additional file 1: Table S1 — Single nucleotide polymorphism (SNP) datasets: replicated loci showing association with oligoarticular and rheumatoid factor-negative (RF-ve) polyarticular juvenile idiopathic arthritis (JIA) were identified from published literature and collated with top genome-wide association studies (GWAS) findings (A). The collated list of genes (B) was used as the seed genes for the network analysis and the generation of the JIA interactome (Figure 1). [file ar4559-S1.doc]

**Supplementary Table 1A**

|  | |  | | |  | | |  | | | |  | |  | |  | |  | |  | |  | |  | |  | |  |  | |  | |  | |  |
| --- | --- | --- | --- | --- | --- | --- | --- | --- | --- | --- | --- | --- | --- | --- | --- | --- | --- | --- | --- | --- | --- | --- | --- | --- | --- | --- | --- | --- | --- | --- | --- | --- | --- | --- | --- |
| **SNP** | | **CHR** | | | **GENES** | | |  | | | |  | |  | |  | | **REF** | |  | |  | |  | |  | |  |  | |  | |  | |  |
| rs2280153 | | 6 | | | LAMA4 | | | TUBE1 | | | | WISP3 | | LOC619208 | |  | | [24] | |  | |  | |  | |  | |  |  | |  | |  | |  |
| rs12046117 | | 1 | | | VTCN1 | | |  | | | |  | |  | |  | | [25] | |  | |  | |  | |  | |  |  | |  | |  | |  |
| rs755622 | | 22 | | | MIF | | |  | | | |  | |  | |  | | [23] | |  | |  | |  | |  | |  |  | |  | |  | |  |
| rs4272626 | | 1 | | | NHLH2 | | |  | | | |  | |  | |  | | [29] | |  | |  | |  | |  | |  |  | |  | |  | |  |
| rs1773560 | | 1 | | | CD247 | | |  | | | |  | |  | |  | |  | |  | |  | |  | |  | |  |  | |  | |  | |  |
| rs10919563 | | 1 | | | PTPRC | | |  | | | |  | |  | |  | |  | |  | |  | |  | |  | |  |  | |  | |  | |  |
| rs706778 | | 10 | | | IL2RA | | |  | | | |  | |  | |  | |  | |  | |  | |  | |  | |  |  | |  | |  | |  |
| rs7234029 | | 18 | | | PTPN2 | | |  | | | |  | |  | |  | |  | |  | |  | |  | |  | |  |  | |  | |  | |  |
| rs8179673 | | 2 | | | STAT1 | | | GLS | | | | STAT4 | |  | |  | | [26] | |  | |  | |  | |  | |  |  | |  | |  | |  |
| rs10181656 | | 2 | | | STAT1 | | | GLS | | | | STAT4 | |  | |  | |  | |  | |  | |  | |  | |  |  | |  | |  | |  |
| rs6920220 | | 6 | | | OLIG3 | | | TNFAIP3 | | | |  | |  | |  | |  | |  | |  | |  | |  | |  |  | |  | |  | |  |
| rs13207033 | | 6 | | | OLIG3 | | | TNFAIP3 | | | |  | |  | |  | |  | |  | |  | |  | |  | |  |  | |  | |  | |  |
| rs2900180 | | 9 | | | PHF19 | | | CEP110 | | | | GSN | | TRAF1 | | RAB14 | | C5 | |  | |  | |  | |  | |  |  | |  | |  | |  |
| rs4750316 | | 10 | | | RBM17 | | | PFKFB3 | | | | PRKCQ | |  | |  | |  | |  | |  | |  | |  | |  |  | |  | |  | |  |
| rs6766899 | | 3 | | | CDGAP | | | C3orf1 | | | | TMEM39A | | CD80 | | KTELC1 | |  | |  | |  | |  | |  | |  |  | |  | |  | |  |
| rs4688011 | | 3 | | | CDGAP | | | C3orf1 | | | | TMEM39A | | CD80 | | KTELC1 | |  | |  | |  | |  | |  | |  |  | |  | |  | |  |
| rs13139573 | | 4 | | | IL15 | | |  | | | |  | |  | |  | |  | |  | |  | |  | |  | |  |  | |  | |  | |  |
| rs4254850 | | 4 | | | IL15 | | |  | | | |  | |  | |  | |  | |  | |  | |  | |  | |  |  | |  | |  | |  |
| rs10995447 | | 10 | | | REEP3 | | | NRBF2 | | | | JMJD1C | |  | |  | |  | |  | |  | |  | |  | |  |  | |  | |  | |  |
| rs10995450 | | 10 | | | REEP3 | | | NRBF2 | | | | JMJD1C | |  | |  | |  | |  | |  | |  | |  | |  |  | |  | |  | |  |
| rs6479891 | | 10 | | | REEP3 | | | NRBF2 | | | | JMJD1C | |  | |  | |  | |  | |  | |  | |  | |  |  | |  | |  | |  |
| rs10761747 | | 10 | | | REEP3 | | | NRBF2 | | | | JMJD1C | |  | |  | |  | |  | |  | |  | |  | |  |  | |  | |  | |  |
| rs12411988 | | 10 | | | REEP3 | | | NRBF2 | | | | JMJD1C | |  | |  | |  | |  | |  | |  | |  | |  |  | |  | |  | |  |
| rs12719740 | | 15 | | | FLJ39743 | | |  | | | |  | |  | |  | |  | |  | |  | |  | |  | |  |  | |  | |  | |  |
| rs2476601 | | 1 | | | PTPN22 | | |  | | | |  | |  | |  | |  | |  | |  | |  | |  | |  |  | |  | |  | |  |
| rs9302588 | | 16 | | | CHD9 | | |  | | | |  | |  | |  | |  | |  | |  | |  | |  | |  |  | |  | |  | |  |
| rs7993214 | | 13 | | | COG6 | | |  | | | |  | |  | |  | |  | |  | |  | |  | |  | |  |  | |  | |  | |  |
| rs6822844 | | 4 | | | IL2 | | | IL21 | | | | ADAD1 | | KIAA1109 | |  | |  | |  | |  | |  | |  | |  |  | |  | |  | |  |
| rs1160542 | | 2 | | | CHST10 | | | LONRF2 | | | | AFF3 | |  | |  | |  | |  | |  | |  | |  | |  |  | |  | |  | |  |
| rs1464510 | | 3 | | | LPP | | |  | | | |  | |  | |  | |  | |  | |  | |  | |  | |  |  | |  | |  | |  |
| **TOP GWAS** | | **p≤1x10^-4^** | | | [6] | | | | | | |  | |  | |  | |  | |  | |  | |  | |  | |  |  | |  | |  | |  |
| rs215792 | | 1 | | | GRIK3 | | |  | | | |  | |  | |  | |  | |  | |  | |  | |  | |  |  | |  | |  | |  |
| rs1288632 | | 1 | | | GLIS1 | | | FLJ40434 | | | | DMRTB1 | |  | |  | |  | |  | |  | |  | |  | |  |  | |  | |  | |  |
| rs1108989 | | 1 | | | GLIS1 | | | FLJ40434 | | | | DMRTB1 | |  | |  | |  | |  | |  | |  | |  | |  |  | |  | |  | |  |
| rs3811438 | | 1 | | | ELTD1 | | |  | | | |  | |  | |  | |  | |  | |  | |  | |  | |  |  | |  | |  | |  |
| rs12029928 | | 1 | | | ELTD1 | | | MAGI3 | | | | BCL2L15 | | PHTF1 | |  | |  | |  | |  | |  | |  | |  |  | |  | |  | |  |
| rs1230666 | | 1 | | | PTPN22 | | | AP4B1 | | | | RSBN1 | | BCL2L15 | | DCLRE1B | | MAGI3 | | BCL2L15 | | PHTF1 | |  | |  | |  |  | |  | |  | |  |
| rs6679677 | | 1 | | | PTPN22 | | | AP4B1 | | | | RSBN1 | | BCL2L15 | | DCLRE1B | | MAGI3 | | BCL2L15 | | PHTF1 | |  | |  | |  |  | |  | |  | |  |
| rs10776775 | | 1 | | | PTPN22 | | | AP4B1 | | | | RSBN1 | | BCL2L15 | | DCLRE1B | |  | |  | |  | |  | |  | |  |  | |  | |  | |  |
| rs10776801 | | 1 | | | VANGL1 | | | CASQ2 | | | | TSPAN2 | | NGF | |  | |  | |  | |  | |  | |  | |  |  | |  | |  | |  |
| rs7511950 | | 1 | | | PLA2G4A | | |  | | | |  | |  | |  | |  | |  | |  | |  | |  | |  |  | |  | |  | |  |
| rs1009221 | | 2 | | | KCNK3 | | |  | | | |  | |  | |  | |  | |  | |  | |  | |  | |  |  | |  | |  | |  |
| rs13427136 | | 2 | | | THUMPD2 | | | TMEM178 | | | | | |  | |  | |  | |  | |  | |  | |  | |  |  | |  | |  | |  |
| rs11690012 | | 2 | | | ZFP36L2 | | | THADA | | | |  | |  | |  | |  | |  | |  | |  | |  | |  |  | |  | |  | |  |
| rs2118480 | | 2 | | | BCL11A | | |  | | | |  | |  | |  | |  | |  | |  | |  | |  | |  |  | |  | |  | |  |
| rs268132 | | 2 | | | SPRED2 | | |  | | | |  | |  | |  | |  | |  | |  | |  | |  | |  |  | |  | |  | |  |
| rs10210235 | | 2 | | | ATP6V1B1 | | |  | | | |  | |  | |  | |  | |  | |  | |  | |  | |  |  | |  | |  | |  |
| rs12987484 | | 2 | | | NCK2 | | |  | | | |  | |  | |  | |  | |  | |  | |  | |  | |  |  | |  | |  | |  |
| rs260705 | | 2 | | | EDAR | | |  | | | |  | |  | |  | |  | |  | |  | |  | |  | |  |  | |  | |  | |  |
| rs13019180 | | 2 | | | DPP10 | | |  | | | |  | |  | |  | |  | |  | |  | |  | |  | |  |  | |  | |  | |  |
| rs13033400 | | 2 | | | DPP10 | | |  | | | |  | |  | |  | |  | |  | |  | |  | |  | |  |  | |  | |  | |  |
| rs1374271 | | 2 | | | DPP10 | | |  | | | |  | |  | |  | |  | |  | |  | |  | |  | |  |  | |  | |  | |  |
| rs997033 | | 2 | | |  | | |  | | | |  | |  | |  | |  | |  | |  | |  | |  | |  |  | |  | |  | |  |
| rs4533492 | | 2 | | |  | | |  | | | |  | |  | |  | |  | |  | |  | |  | |  | |  |  | |  | |  | |  |
| rs330646 | | 2 | | |  | | |  | | | |  | |  | |  | |  | |  | |  | |  | |  | |  |  | |  | |  | |  |
| rs330625 | | 2 | | |  | | |  | | | |  | |  | |  | |  | |  | |  | |  | |  | |  |  | |  | |  | |  |
| rs108136 | | 2 | | |  | | |  | | | |  | |  | |  | |  | |  | |  | |  | |  | |  |  | |  | |  | |  |
| rs330624 | | 2 | | |  | | |  | | | |  | |  | |  | |  | |  | |  | |  | |  | |  |  | |  | |  | |  |
| rs10931480 | | 2 | | | STAT1 | | | GLS | | | | STAT4 | |  | |  | |  | |  | |  | |  | |  | |  |  | |  | |  | |  |
| rs12466358 | | 2 | | | TIGD1 | | | CHRNG | | | | EIF4E2 | | CHRND | |  | |  | |  | |  | |  | |  | |  |  | |  | |  | |  |
| rs11894280 | | 2 | | | RAB17 | | | LRRFIP1 | | | |  | |  | |  | |  | |  | |  | |  | |  | |  |  | |  | |  | |  |
| rs12464181 | | 2 | | | RAB17 | | | LRRFIP1 | | | |  | |  | |  | |  | |  | |  | |  | |  | |  |  | |  | |  | |  |
| rs12464145 | | 2 | | | RAB17 | | | LRRFIP1 | | | |  | |  | |  | |  | |  | |  | |  | |  | |  |  | |  | |  | |  |
| rs4663779 | | 2 | | | LRRFIP1 | | |  | | | |  | |  | |  | |  | |  | |  | |  | |  | |  |  | |  | |  | |  |
| rs1552484 | | 3 | | | RAF1 | | | CAND2 | | | | TSEN2 | | TMEM40 | | MKRN2 | |  | |  | |  | |  | |  | |  |  | |  | |  | |  |
| rs2596822 | | 3 | | | RAF1 | | | CAND2 | | | | TSEN2 | | TMEM40 | | MKRN2 | |  | |  | |  | |  | |  | |  |  | |  | |  | |  |
| rs2454422 | | 3 | | | RAF1 | | | CAND2 | | | | TSEN2 | | TMEM40 | | MKRN2 | |  | |  | |  | |  | |  | |  |  | |  | |  | |  |
| rs1532533 | | 3 | | | RAF1 | | | CAND2 | | | | TSEN2 | | TMEM40 | | MKRN2 | |  | |  | |  | |  | |  | |  |  | |  | |  | |  |
| rs6781484 | | 3 | | | FBXL2 | | | UBP1 | | | | CLASP2 | |  | |  | |  | |  | |  | |  | |  | |  |  | |  | |  | |  |
| rs8179967 | | 3 | | | FBXL2 | | | UBP1 | | | | CLASP2 | |  | |  | |  | |  | |  | |  | |  | |  |  | |  | |  | |  |
| rs6807542 | | 3 | | | FBXL2 | | | UBP1 | | | | CLASP2 | |  | |  | |  | |  | |  | |  | |  | |  |  | |  | |  | |  |
| rs11707471 | | 3 | | | CCR5 | | | LTF | | | | FLJ78302 | |  | |  | |  | |  | |  | |  | |  | |  |  | |  | |  | |  |
| rs771798 | | 3 | | | CEP97 | | | LOC285359 | | | | NFKBIZ | | ZPLD1 | | FAM55C | |  | |  | |  | |  | |  | |  |  | |  | |  | |  |
| rs771795 | | 3 | | | CEP97 | | | LOC285359 | | | | NFKBIZ | | ZPLD1 | | FAM55C | |  | |  | |  | |  | |  | |  |  | |  | |  | |  |
| rs771797 | | 3 | | | CEP97 | | | LOC285359 | | | | NFKBIZ | | ZPLD1 | | FAM55C | |  | |  | |  | |  | |  | |  |  | |  | |  | |  |
| rs4683863 | | 3 | | | CEP97 | | | LOC285359 | | | | NFKBIZ | | ZPLD1 | | FAM55C | |  | |  | |  | |  | |  | |  |  | |  | |  | |  |
| rs3755579 | | 3 | | | CDGAP | | | C3orf1 | | | | TMEM39A | | CD80 | | KTELC1 | |  | |  | |  | |  | |  | |  |  | |  | |  | |  |
| rs4447803 | | 3 | | | CDGAP | | | C3orf1 | | | | TMEM39A | | CD80 | | KTELC1 | |  | |  | |  | |  | |  | |  |  | |  | |  | |  |
| rs4461452 | | 3 | | | CDGAP | | | C3orf1 | | | | TMEM39A | | CD80 | | KTELC1 | |  | |  | |  | |  | |  | |  |  | |  | |  | |  |
| rs2886059 | | 3 | | | ALDH1L1 | | |  | | | |  | |  | |  | |  | |  | |  | |  | |  | |  |  | |  | |  | |  |
| rs541042 | | 3 | | | MGLL | | |  | | | |  | |  | |  | |  | |  | |  | |  | |  | |  |  | |  | |  | |  |
| rs838601 | | 3 | | | SLC9A9 | | |  | | | |  | |  | |  | |  | |  | |  | |  | |  | |  |  | |  | |  | |  |
| rs7646910 | | 3 | | | EPHB3 | | | VPS8 | | | | MAGEF1 | | CHRD | |  | |  | |  | |  | |  | |  | |  |  | |  | |  | |  |
| rs4686705 | | 3 | | | EPHB3 | | | VPS8 | | | | MAGEF1 | | CHRD | |  | |  | |  | |  | |  | |  | |  |  | |  | |  | |  |
| rs1947549 | | 4 | | |  | | |  | | | |  | |  | |  | |  | |  | |  | |  | |  | |  |  | |  | |  | |  |
| rs6815080 | | 4 | | | EPHA5 | | |  | | | |  | |  | |  | |  | |  | |  | |  | |  | |  |  | |  | |  | |  |
| rs7690697 | | 4 | | | ANTXR2 | | |  | | | |  | |  | |  | |  | |  | |  | |  | |  | |  |  | |  | |  | |  |
| rs4234847 | | 4 | | | ANTXR2 | | |  | | | |  | |  | |  | |  | |  | |  | |  | |  | |  |  | |  | |  | |  |
| rs10034564 | | 4 | | |  | | |  | | | |  | |  | |  | |  | |  | |  | |  | |  | |  |  | |  | |  | |  |
| rs12499753 | | 4 | | | IL2 | | | IL21 | | | | ADAD1 | | ADAD1 | | KIAA1109 | |  | |  | |  | |  | |  | |  |  | |  | |  | |  |
| rs17388568 | | 4 | | | IL2 | | | IL21 | | | | ADAD1 | | ADAD1 | | KIAA1109 | |  | |  | |  | |  | |  | |  |  | |  | |  | |  |
| rs11724582 | | 4 | | | IL2 | | | IL21 | | | | ADAD1 | | ADAD1 | | KIAA1109 | |  | |  | |  | |  | |  | |  |  | |  | |  | |  |
| rs11098659 | | 4 | | | IL2 | | | IL21 | | | | ADAD1 | | ADAD1 | | KIAA1109 | |  | |  | |  | |  | |  | |  |  | |  | |  | |  |
| rs6829845 | | 4 | | | IL2 | | | IL21 | | | | ADAD1 | | ADAD1 | | KIAA1109 | |  | |  | |  | |  | |  | |  |  | |  | |  | |  |
| rs925549 | | 4 | | | IL2 | | | IL21 | | | | ADAD1 | | ADAD1 | | KIAA1109 | |  | |  | |  | |  | |  | |  |  | |  | |  | |  |
| rs2322305 | | 4 | | | IL15 | | |  | | | |  | |  | |  | |  | |  | |  | |  | |  | |  |  | |  | |  | |  |
| rs1602777 | | 4 | | | IL15 | | |  | | | |  | |  | |  | |  | |  | |  | |  | |  | |  |  | |  | |  | |  |
| rs970509 | | 4 | | | IL15 | | |  | | | |  | |  | |  | |  | |  | |  | |  | |  | |  |  | |  | |  | |  |
| rs11724130 | | 4 | | |  | | |  | | | |  | |  | |  | |  | |  | |  | |  | |  | |  |  | |  | |  | |  |
| rs1125051 | | 4 | | |  | | |  | | | |  | |  | |  | |  | |  | |  | |  | |  | |  |  | |  | |  | |  |
| rs6535695 | | 4 | | |  | | |  | | | |  | |  | |  | |  | |  | |  | |  | |  | |  |  | |  | |  | |  |
| rs10520942 | | 5 | | |  | | |  | | | |  | |  | |  | |  | |  | |  | |  | |  | |  |  | |  | |  | |  |
| rs283595 | | 5 | | | RGNEF | | |  | | | |  | |  | |  | |  | |  | |  | |  | |  | |  |  | |  | |  | |  |
| rs11748911 | | 5 | | | GPR98 | | | POLR3G | | | | LYSMD3 | |  | |  | |  | |  | |  | |  | |  | |  |  | |  | |  | |  |
| rs1428638 | | 5 | | | DTWD2 | | |  | | | |  | |  | |  | |  | |  | |  | |  | |  | |  |  | |  | |  | |  |
| rs2548997 | | 5 | | | LOC441108 | | | IRF1 | | | |  | |  | |  | |  | |  | |  | |  | |  | |  |  | |  | |  | |  |
| rs949637 | | 5 | | | MAPK9 | | |  | | | |  | |  | |  | |  | |  | |  | |  | |  | |  |  | |  | |  | |  |
| rs4700921 | | 5 | | | SCGB3A1 | | | CNOT6 | | | | RASGEF1C | | GFPT2 | | FLT4 | | MAPK9 | |  | |  | |  | |  | |  |  | |  | |  | |  |
| rs17080306 | | 5 | | | SCGB3A1 | | | CNOT6 | | | | RASGEF1C | | GFPT2 | | FLT4 | | MAPK9 | |  | |  | |  | |  | |  |  | |  | |  | |  |
| rs17629918 | | 5 | | | SCGB3A1 | | | CNOT6 | | | | RASGEF1C | | GFPT2 | | FLT4 | |  | |  | |  | |  | |  | |  |  | |  | |  | |  |
| rs12191519 | | 6 | | | IL17F | | | MCM3 | | | |  | |  | |  | |  | |  | |  | |  | |  | |  |  | |  | |  | |  |
| rs7772171 | | 6 | | | ATG5 | | | PRDM1 | | | |  | |  | |  | |  | |  | |  | |  | |  | |  |  | |  | |  | |  |
| rs633724 | | 6 | | | ATG5 | | |  | | | |  | |  | |  | |  | |  | |  | |  | |  | |  |  | |  | |  | |  |
| rs533685 | | 6 | | | ATG5 | | |  | | | |  | |  | |  | |  | |  | |  | |  | |  | |  |  | |  | |  | |  |
| rs3862834 | | 6 | | | SCML4 | | |  | | | |  | |  | |  | |  | |  | |  | |  | |  | |  |  | |  | |  | |  |
| rs1624055 | | 6 | | | SCML4 | | |  | | | |  | |  | |  | |  | |  | |  | |  | |  | |  |  | |  | |  | |  |
| rs1629910 | | 6 | | | SCML4 | | |  | | | |  | |  | |  | |  | |  | |  | |  | |  | |  |  | |  | |  | |  |
| rs1621495 | | 6 | | | SCML4 | | |  | | | |  | |  | |  | |  | |  | |  | |  | |  | |  |  | |  | |  | |  |
| rs1728123 | | 6 | | | SCML4 | | |  | | | |  | |  | |  | |  | |  | |  | |  | |  | |  |  | |  | |  | |  |
| rs6922304 | | 6 | | | SCML4 | | |  | | | |  | |  | |  | |  | |  | |  | |  | |  | |  |  | |  | |  | |  |
| rs9320231 | | 6 | | | SCML4 | | |  | | | |  | |  | |  | |  | |  | |  | |  | |  | |  |  | |  | |  | |  |
| rs12664414 | | 6 | | | SCML4 | | |  | | | |  | |  | |  | |  | |  | |  | |  | |  | |  |  | |  | |  | |  |
| rs1321270 | | 6 | | | CTGF | | | MOXD1 | | | | ENPP1 | |  | |  | |  | |  | |  | |  | |  | |  |  | |  | |  | |  |
| rs2614264 | | 6 | | | AHI1 | | |  | | | |  | |  | |  | |  | |  | |  | |  | |  | |  |  | |  | |  | |  |
| rs6463967 | | 7 | | |  | | |  | | | |  | |  | |  | |  | |  | |  | |  | |  | |  |  | |  | |  | |  |
| rs17143897 | | 7 | | | SP8 | | |  | | | |  | |  | |  | |  | |  | |  | |  | |  | |  |  | |  | |  | |  |
| rs3173734 | | 7 | | | LOC441208 | | | LSM5 | | | | KIAA0241 | |  | |  | |  | |  | |  | |  | |  | |  |  | |  | |  | |  |
| rs2621208 | | 7 | | | COL1A2 | | |  | | | |  | |  | |  | |  | |  | |  | |  | |  | |  |  | |  | |  | |  |
| rs4729451 | | 7 | | | NPTX2 | | | DKFZP434B0335 | | | | BAIAP2L1 | | BRI3 | |  | |  | |  | |  | |  | |  | |  |  | |  | |  | |  |
| rs390547 | | 7 | | | LAMB4 | | |  | | | |  | |  | |  | |  | |  | |  | |  | |  | |  |  | |  | |  | |  |
| rs1550950 | | 8 | | | FBXO25 | | | C8orf42 | | | |  | |  | |  | |  | |  | |  | |  | |  | |  |  | |  | |  | |  |
| rs1550948 | | 8 | | | FBXO25 | | | C8orf42 | | | |  | |  | |  | |  | |  | |  | |  | |  | |  |  | |  | |  | |  |
| rs4871834 | | 8 | | | PEBP4 | | |  | | | |  | |  | |  | |  | |  | |  | |  | |  | |  |  | |  | |  | |  |
| rs7836768 | | 8 | | | BHLHB5 | | | LOC401463 | | | | CYP7B1 | |  | |  | |  | |  | |  | |  | |  | |  |  | |  | |  | |  |
| rs381349 | | 8 | | | hCG_1984468 | | | ZNF704 | | | | ZBTB10 | |  | |  | |  | |  | |  | |  | |  | |  |  | |  | |  | |  |
| rs1788148 | | 8 | | | STK3 | | | VPS13B | | | | OSR2 | |  | |  | |  | |  | |  | |  | |  | |  |  | |  | |  | |  |
| rs678839 | | 8 | | | AZIN1 | | |  | | | |  | |  | |  | |  | |  | |  | |  | |  | |  |  | |  | |  | |  |
| rs1532560 | | 8 | | | PVT1 | | |  | | | |  | |  | |  | |  | |  | |  | |  | |  | |  |  | |  | |  | |  |
| rs12002949 | | 9 | | |  | | |  | | | |  | |  | |  | |  | |  | |  | |  | |  | |  |  | |  | |  | |  |
| rs1536689 | | 9 | | | C9orf93 | | | LOC730112 | | | | UNC13B | |  | |  | |  | |  | |  | |  | |  | |  |  | |  | |  | |  |
| rs10814234 | | 9 | | | CD72 | | | RUSC2 | | | | LOC158381 | | SIT1 | | TESK1 | |  | |  | |  | |  | |  | |  |  | |  | |  | |  |
| rs10735544 | | 9 | | |  | | |  | | | |  | |  | |  | |  | |  | |  | |  | |  | |  |  | |  | |  | |  |
| rs12686543 | | 9 | | |  | | |  | | | |  | |  | |  | |  | |  | |  | |  | |  | |  |  | |  | |  | |  |
| rs7851576 | | 9 | | |  | | |  | | | |  | |  | |  | |  | |  | |  | |  | |  | |  |  | |  | |  | |  |
| rs4877557 | | 9 | | |  | | |  | | | |  | |  | |  | |  | |  | |  | |  | |  | |  |  | |  | |  | |  |
| rs11250787 | | 10 | | | C10orf109 | | | ADARB2 | | | |  | |  | |  | |  | |  | |  | |  | |  | |  |  | |  | |  | |  |
| rs4367871 | | 10 | | | C10orf109 | | | ADARB2 | | | |  | |  | |  | |  | |  | |  | |  | |  | |  |  | |  | |  | |  |
| rs10795791 | | 10 | | | IL2RA | | |  | | | |  | |  | |  | |  | |  | |  | |  | |  | |  |  | |  | |  | |  |
| rs1999241 | | 10 | | | MYO3A | | | GAD2 | | | |  | |  | |  | |  | |  | |  | |  | |  | |  |  | |  | |  | |  |
| rs16917442 | | 10 | | | ZNF365 | | |  | | | |  | |  | |  | |  | |  | |  | |  | |  | |  |  | |  | |  | |  |
| rs12416113 | | 10 | | | REEP3 | | | NRBF2 | | | | JMJD1C | |  | |  | |  | |  | |  | |  | |  | |  |  | |  | |  | |  |
| rs11817689 | | 10 | | | REEP3 | | | NRBF2 | | | | JMJD1C | |  | |  | |  | |  | |  | |  | |  | |  |  | |  | |  | |  |
| rs1009984 | | 10 | | | REEP3 | | | NRBF2 | | | | JMJD1C | |  | |  | |  | |  | |  | |  | |  | |  |  | |  | |  | |  |
| rs7082090 | | 10 | | | REEP3 | | | NRBF2 | | | | JMJD1C | |  | |  | |  | |  | |  | |  | |  | |  |  | |  | |  | |  |
| rs9414780 | | 10 | | | REEP3 | | | NRBF2 | | | | JMJD1C | |  | |  | |  | |  | |  | |  | |  | |  |  | |  | |  | |  |
| rs9414788 | | 10 | | | REEP3 | | | NRBF2 | | | | JMJD1C | |  | |  | |  | |  | |  | |  | |  | |  |  | |  | |  | |  |
| rs10761745 | | 10 | | | REEP3 | | | NRBF2 | | | | JMJD1C | |  | |  | |  | |  | |  | |  | |  | |  |  | |  | |  | |  |
| rs10761744 | | 10 | | | REEP3 | | | NRBF2 | | | | JMJD1C | |  | |  | |  | |  | |  | |  | |  | |  |  | |  | |  | |  |
| rs9415699 | | 10 | | | REEP3 | | | NRBF2 | | | | JMJD1C | |  | |  | |  | |  | |  | |  | |  | |  |  | |  | |  | |  |
| rs9415705 | | 10 | | | REEP3 | | | NRBF2 | | | | JMJD1C | |  | |  | |  | |  | |  | |  | |  | |  |  | |  | |  | |  |
| rs2893922 | | 10 | | | REEP3 | | | NRBF2 | | | | JMJD1C | |  | |  | |  | |  | |  | |  | |  | |  |  | |  | |  | |  |
| rs10761781 | | 10 | | | REEP3 | | | NRBF2 | | | | JMJD1C | |  | |  | |  | |  | |  | |  | |  | |  |  | |  | |  | |  |
| rs16918575 | | 10 | | | REEP3 | | | NRBF2 | | | | JMJD1C | |  | |  | |  | |  | |  | |  | |  | |  |  | |  | |  | |  |
| rs4746201 | | 10 | | | REEP3 | | | NRBF2 | | | | JMJD1C | |  | |  | |  | |  | |  | |  | |  | |  |  | |  | |  | |  |
| rs1426619 | | 10 | | | C10orf59 | | |  | | | |  | |  | |  | |  | |  | |  | |  | |  | |  |  | |  | |  | |  |
| rs10882303 | | 10 | | | PDE6C | | | C10orf4 | | | | LGI1 | | ENTPD1 | |  | |  | |  | |  | |  | |  | |  |  | |  | |  | |  |
| rs11188409 | | 10 | | | TCTN3 | | | CC2D2B | | | | ALDH18A1 | | TCTN3 | | SORBS1 | |  | |  | |  | |  | |  | |  |  | |  | |  | |  |
| rs4453231 | | 11 | | | GALNTL4 | | |  | | | |  | |  | |  | |  | |  | |  | |  | |  | |  |  | |  | |  | |  |
| rs10832890 | | 11 | | | MRGPRX4 | | |  | | | |  | |  | |  | |  | |  | |  | |  | |  | |  |  | |  | |  | |  |
| rs2863140 | | 11 | | | PRDM11 | | | TP53I11 | | | | SYT13 | | TSPAN18 | | LRP4 | | AMBRA1 | | C11orf77 | | KIAA0652 | | CKAP5 | | AMBRA1 | | F2 | SNORD67 | | DGKZ | |  | |  |
| rs12283172 | | 11 | | | ZNF408 | | | CHRM4 | | | | CREB3L1 | | ARHGAP1 | | MDK | |  | |  | |  | |  | |  | |  |  | |  | |  | |  |
| rs6489438 | | 12 | | | TSPAN9 | | |  | | | |  | |  | |  | |  | |  | |  | |  | |  | |  |  | |  | |  | |  |
| rs11047723 | | 12 | | | BCAT1 | | |  | | | |  | |  | |  | |  | |  | |  | |  | |  | |  |  | |  | |  | |  |
| rs10880908 | | 12 | | | ARID2 | | | SFRS2IP | | | | SLC38A2 | | SLC38A1 | |  | |  | |  | |  | |  | |  | |  |  | |  | |  | |  |
| rs872969 | | 12 | | | CLLU1 | | | BTG1 | | | | CLLU1OS | |  | |  | |  | |  | |  | |  | |  | |  |  | |  | |  | |  |
| rs11059562 | | 12 | | |  | | |  | | | |  | |  | |  | |  | |  | |  | |  | |  | |  |  | |  | |  | |  |
| rs4941939 | | 13 | | | COG6 | | |  | | | |  | |  | |  | |  | |  | |  | |  | |  | |  |  | |  | |  | |  |
| rs17061417 | | 13 | | |  | | |  | | | |  | |  | |  | |  | |  | |  | |  | |  | |  |  | |  | |  | |  |
| rs17063408 | | 13 | | | SUPT16H | | | RNASE8 | | | | NDRG2 | | METT11D1 | | ZNF219 | | SNORD8 | | FLJ10357 | | HNRNPC | | CHD8 | | OR5AU1 | | TPPP2 | SNORD9 | |  | |  | |  |
| rs17102539 | | 14 | | | SLC39A2 | | | RNASE7 | | | | RNASE2 | | RNASE13 | | RPGRIP1 | |  | |  | |  | |  | |  | |  |  | |  | |  | |  |
| rs8020810 | | 14 | | | STXBP6 | | |  | | | |  | |  | |  | |  | |  | |  | |  | |  | |  |  | |  | |  | |  |
| rs7359140 | | 14 | | | JDP2 | | |  | | | |  | |  | |  | |  | |  | |  | |  | |  | |  |  | |  | |  | |  |
| rs7359152 | | 14 | | | JDP2 | | |  | | | |  | |  | |  | |  | |  | |  | |  | |  | |  |  | |  | |  | |  |
| rs8043332 | | 15 | | | FLJ39743 | | |  | | | |  | |  | |  | |  | |  | |  | |  | |  | |  |  | |  | |  | |  |
| rs11074618 | | 16 | | | CACNG3 | | |  | | | |  | |  | |  | |  | |  | |  | |  | |  | |  |  | |  | |  | |  |
| rs9936233 | | 16 | | | CACNG3 | | |  | | | |  | |  | |  | |  | |  | |  | |  | |  | |  |  | |  | |  | |  |
| rs9930106 | | 16 | | | CHD9 | | | GCSH | | | | CENPN | |  | |  | |  | |  | |  | |  | |  | |  |  | |  | |  | |  |
| rs2432414 | | 16 | | | ATMIN | | | C16orf46 | | | | PKD1L2 | | CDYL2 | | C16orf61 | |  | |  | |  | |  | |  | |  |  | |  | |  | |  |
| rs4790365 | | 17 | | | GARNL4 | | |  | | | |  | |  | |  | |  | |  | |  | |  | |  | |  |  | |  | |  | |  |
| rs11653603 | | 17 | | | MYH13 | | |  | | | |  | |  | |  | |  | |  | |  | |  | |  | |  |  | |  | |  | |  |
| rs12950258 | | 17 | | | MYH13 | | |  | | | |  | |  | |  | |  | |  | |  | |  | |  | |  |  | |  | |  | |  |
| rs11658620 | | 17 | | | MYH13 | | |  | | | |  | |  | |  | |  | |  | |  | |  | |  | |  |  | |  | |  | |  |
| rs12951092 | | 17 | | | MYH13 | | |  | | | |  | |  | |  | |  | |  | |  | |  | |  | |  |  | |  | |  | |  |
| rs10521213 | | 17 | | | ELAC2 | | | RICH2 | | | | HS3ST3A1 | |  | |  | |  | |  | |  | |  | |  | |  |  | |  | |  | |  |
| rs230922 | | 17 | | | PMP22 | | |  | | | |  | |  | |  | |  | |  | |  | |  | |  | |  |  | |  | |  | |  |
| rs230915 | | 17 | | | PMP22 | | |  | | | |  | |  | |  | |  | |  | |  | |  | |  | |  |  | |  | |  | |  |
| rs9901734 | | 17 | | | KSR1 | | | LGALS9 | | | | NOS2A | | TMEM49 | | PTRH2 | |  | |  | |  | |  | |  | |  |  | |  | |  | |  |
| rs2526354 | | 17 | | | CLTC | | | RPS6KB1 | | | | RNFT1 | | DHX40 | | TUBD1 | |  | |  | |  | |  | |  | |  |  | |  | |  | |  |
| rs11659412 | | 18 | | | LAMA1 | | |  | | | |  | |  | |  | |  | |  | |  | |  | |  | |  |  | |  | |  | |  |
| rs2542151 | | 18 | | | PTPN2 | | |  | | | |  | |  | |  | |  | |  | |  | |  | |  | |  |  | |  | |  | |  |
| rs2542160 | | 18 | | | PTPN2 | | |  | | | |  | |  | |  | |  | |  | |  | |  | |  | |  |  | |  | |  | |  |
| rs2847297 | | 18 | | | PTPN2 | | |  | | | |  | |  | |  | |  | |  | |  | |  | |  | |  |  | |  | |  | |  |
| rs16939895 | | 18 | | | PTPN2 | | |  | | | |  | |  | |  | |  | |  | |  | |  | |  | |  |  | |  | |  | |  |
| rs2847281 | | 18 | | | PTPN2 | | |  | | | |  | |  | |  | |  | |  | |  | |  | |  | |  |  | |  | |  | |  |
| rs8087237 | | 18 | | | PTPN2 | | |  | | | |  | |  | |  | |  | |  | |  | |  | |  | |  |  | |  | |  | |  |
| rs7234029 | | 18 | | | PTPN2 | | |  | | | |  | |  | |  | |  | |  | |  | |  | |  | |  |  | |  | |  | |  |
| rs12968853 | | 18 | | | SNORD58C | | | SCARNA17 | | | | LIPG | | RPL17 | | C18orf32 | |  | |  | |  | |  | |  | |  |  | |  | |  | |  |
| rs12970803 | | 18 | | | ACAA2 | | | DYM | | | | SNORD58A | | SNORD58B | | MYO5B | |  | |  | |  | |  | |  | |  |  | |  | |  | |  |
| rs6507952 | | 18 | | | ACAA2 | | | MYO5B | | | | SCARNA17 | |  | |  | |  | |  | |  | |  | |  | |  |  | |  | |  | |  |
| rs7241641 | | 18 | | | ACAA2 | | | MYO5B | | | | SCARNA17 | |  | |  | |  | |  | |  | |  | |  | |  |  | |  | |  | |  |
| rs7237886 | | 18 | | | ZNF516 | | |  | | | |  | |  | |  | |  | |  | |  | |  | |  | |  |  | |  | |  | |  |
| rs4267414 | | 18 | | | ZNF516 | | |  | | | |  | |  | |  | |  | |  | |  | |  | |  | |  |  | |  | |  | |  |
| rs2230611 | | 19 | | | FUT6 | | | PTPRS | | | |  | |  | |  | |  | |  | |  | |  | |  | |  |  | |  | |  | |  |
| rs10419891 | | 19 | | | ZNF415 | | | ZNF160 | | | |  | |  | |  | |  | |  | |  | |  | |  | |  |  | |  | |  | |  |
| rs7255074 | | 19 | | | LOC91664 | | | ZNF525 | | | |  | |  | |  | |  | |  | |  | |  | |  | |  |  | |  | |  | |  |
| rs652325 | | 20 | | | C20orf94 | | | JAG1 | | | |  | |  | |  | |  | |  | |  | |  | |  | |  |  | |  | |  | |  |
| rs622293 | | 20 | | | C20orf94 | | | JAG1 | | | | BCL2L1 | | C20orf191 | | TPX2 | | ID1 | | FRG1B | | FKHL18 | | PSIMCT-1 | | COX4I2 | | ABHD12 | DEFB124 | | DEFB122 | | REM1 | | DEFB118 |
|  | |  | | | GINS1 | | | DEFB123 | | | | PYGB | | DUSP15 | | DEFB121 | | ZNF337 | | DEFB116 | | HM13 | |  | |  | |  |  | |  | |  | |  |
| rs6058022 | | 20 | | | DEFB115 | | | RP4-691N24.1 | | | | NANP | | DEFB119 | | MYLK2 | | BCL2L1 | | C20orf191 | | TPX2 | | ID1 | | FRG1B | | FKHL18 | PSIMCT-1 | | COX4I2 | | ABHD12 | | DEFB124 |
|  | |  | | | DEFB122 | | | REM1 | | | | DEFB118 | | GINS1 | | DEFB123 | | PYGB | | DUSP15 | | DEFB121 | | ZNF337 | | DEFB116 | | HM13 |  | |  | |  | |  |
| rs6059980 | | 20 | | | DEFB115 | | | RP4-691N24.1 | | | | NANP | | DEFB119 | | MYLK2 | | BCL2L1 | | C20orf191 | | TPX2 | | ID1 | | FRG1B | | FKHL18 | PSIMCT-1 | | COX4I2 | | ABHD12 | | DEFB124 |
|  | |  | | | DEFB122 | | | REM1 | | | | DEFB118 | | GINS1 | | DEFB123 | | PYGB | | DUSP15 | | DEFB121 | | ZNF337 | | DEFB116 | | HM13 |  | |  | |  | |  |
| rs6120719 | | 20 | | | DEFB115 | | | RP4-691N24.1 | | | | NANP | | DEFB119 | | MYLK2 | | BCL2L1 | | C20orf191 | | TPX2 | | ID1 | | FRG1B | | FKHL18 | PSIMCT-1 | | COX4I2 | | ABHD12 | | DEFB124 |
|  | |  | | | DEFB122 | | | REM1 | | | | DEFB118 | | GINS1 | | DEFB123 | | PYGB | | DUSP15 | | DEFB121 | | ZNF337 | | DEFB116 | | HM13 |  | |  | |  | |  |
| rs11906851 | | 20 | | | DEFB115 | | | RP4-691N24.1 | | | | NANP | | DEFB119 | | MYLK2 | |  | |  | |  | |  | |  | |  |  | |  | |  | |  |
| rs6126970 | | 20 | | | BCAS1 | | | ZNF217 | | | | SUMO1P1 | |  | |  | |  | |  | |  | |  | |  | |  |  | |  | |  | |  |
| rs6025837 | | 20 | | | TMEPAI | | | ZBP1 | | | |  | |  | |  | |  | |  | |  | |  | |  | |  |  | |  | |  | |  |
| rs2834890 | | 21 | | | RUNX1 | | |  | | | |  | |  | |  | |  | |  | |  | |  | |  | |  |  | |  | |  | |  |
| rs2837193 | | 21 | | | IGSF5 | | | IGSF5 | | | |  | |  | |  | |  | |  | |  | |  | |  | |  |  | |  | |  | |  |
| rs462782 | | 21 | | | IGSF5 | | | IGSF5 | | | |  | |  | |  | |  | |  | |  | |  | |  | |  |  | |  | |  | |  |
| rs458297 | | 21 | | | IGSF5 | | | IGSF5 | | | |  | |  | |  | |  | |  | |  | |  | |  | |  |  | |  | |  | |  |
| rs380673 | | 21 | | | IGSF5 | | | IGSF5 | | | | CHEK2 | | TTC28 | | CCDC117 | |  | |  | |  | |  | |  | |  |  | |  | |  | |  |
| rs1534884 | | 22 | | | ZNRF3 | | | C22orf31 | | | | HSCB | | XBP1 | | PITPNB | | CHEK2 | |  | |  | |  | |  | |  |  | |  | |  | |  |
| rs6005975 | | 22 | | | ZNRF3 | | | C22orf31 | | | | CCDC117 | | HSCB | | XBP1 | |  | |  | |  | |  | |  | |  |  | |  | |  | |  |
| rs5999854 | | 22 | | | MCM5 | | |  | | | |  | |  | |  | |  | |  | |  | |  | |  | |  |  | |  | |  | |  |
| Immunochip loci | | | | | [7] | | |  | | | |  | |  | |  | |  | |  | |  | |  | |  | |  |  | |  | |  | |  |
|  | rs10174238 | 2 | STAT1 | | | | GLS | | | | STAT4 | |  | |  | |  | |  | |  | |  | |  | |  | | |  | |  | |  | |
|  | rs11265608 | 1 | AQP10 | | | | IL6R | | | | ATP8B2 | |  | |  | |  | |  | |  | |  | |  | |  | | |  | |  | |  | |
|  | rs12434551 | 14 | ZFP36L1 | | | | C14orf181 | | | | C14orf181 | | RAD51L1 | |  | |  | |  | |  | |  | |  | |  | | |  | |  | |  | |
|  | rs1479924 | 4 | IL2 | | | | IL21 | | | | ADAD1 | | ADAD1 | | KIAA1109 | |  | |  | |  | |  | |  | |  | | |  | |  | |  | |
|  | rs2266959 | 22 | CCDC116 | | | | UBE2L3 | | | | PI4KAP2 | | HIC2 | | LOC150223 | | | |  | |  | |  | |  | |  | | |  | |  | |  | |
|  | rs2284033 | 22 | IL2RB | | | |  | | | |  | |  | |  | |  | |  | |  | |  | |  | |  | | |  | |  | |  | |
|  | rs27290 | 5 | LNPEP | | | | ERAP2 | | | | ERAP2 | |  | |  | |  | |  | |  | |  | |  | |  | | |  | |  | |  | |
|  | rs3184504 | 12 | ATXN2 | | | | SH2B3 | | | | C12orf30 | | RPL6 | | ERP29 | | TRAFD1 | | PTPN11 | | ACAD10 | | BRAP | | MAPKAPK5 | | C12orf51 | | | ALDH2 | |  | |  | |
|  | rs3825568 | 14 | ZFP36L1 | | | | C14orf181 | | | | C14orf181 | | RAD51L1 | |  | |  | |  | |  | |  | |  | |  | | |  | |  | |  | |
|  | rs4705862 | 5 | SLC22A4 | | | | C5orf56 | | | | SLC22A5 | | IRF1 | |  | |  | |  | |  | |  | |  | |  | | |  | |  | |  | |
|  | rs6679677 | 1 | PTPN22 | | | | AP4B1 | | | | RSBN1 | | BCL2L15 | | DCLRE1B | | MAGI3 | | BCL2L15 | | PHTF1 | |  | |  | |  | | |  | |  | |  | |
|  | rs6894249 | 5 | SLC22A4 | | | | C5orf56 | | | | SLC22A5 | | IRF1 | |  | |  | |  | |  | |  | |  | |  | | |  | |  | |  | |
|  | rs7069750 | 10 | FAS | | | | ACTA2 | | | |  | |  | |  | |  | |  | |  | |  | |  | |  | | |  | |  | |  | |
|  | rs7775055 | 6 | C6orf10 | | | | HLA-DOB | | | | HLA-DOB | | HLA-DQB1 | | BTNL2 | | HLA-DQA1 | | HLA-DRB5 | | HLA-DRB1 | | TAP2 | | HLA-DRA | | HLA-DRB6 | | | HLA-DQB2 | | HLA-DQA2 | |  | |
|  | rs8129030 | 21 | RUNX1 | | | |  | | | |  | |  | |  | |  | |  | |  | |  | |  | |  | | |  | |  | |  | |
|  | rs9979383 | 21 | RUNX1 | | | |  | | | |  | |  | |  | |  | |  | |  | |  | |  | |  | | |  | |  | |  | |
|  | rs2364480 | 12 | SCNN1A | | | | LTBR | | | |  | |  | |  | |  | |  | |  | |  | |  | |  | | |  | |  | |  | |
|  | rs4648881 | 1 | SRRM1 | | | | RUNX3 | | | | CLIC4 | |  | |  | |  | |  | |  | |  | |  | |  | | |  | |  | |  | |
|  | rs4688013 | 3 | CDGAP | | | | C3orf1 | | | | TMEM39A | | CD80 | | KTELC1 | |  | |  | |  | |  | |  | |  | | |  | |  | |  | |
|  | rs4755450 | 11 | COMMD9 | | | | FLJ14213 | | | |  | |  | |  | |  | |  | |  | |  | |  | |  | | |  | |  | |  | |
|  | rs6740838 | 2 | CHST10 | | | | LONRF2 | | | | AFF3 | |  | |  | |  | |  | |  | |  | |  | |  | | |  | |  | |  | |
|  | rs6946509 | 7 | IL6 | | | |  | | | |  | |  | |  | |  | |  | |  | |  | |  | |  | | |  | |  | |  | |
|  | rs7127214 | 11 | COMMD9 | | | | FLJ14213 | | | |  | |  | |  | |  | |  | |  | |  | |  | |  | | |  | |  | |  | |
|  | rs7808122 | 7 | IL6 | | | |  | | | |  | |  | |  | |  | |  | |  | |  | |  | |  | | |  | |  | |  | |
|  | rs7993214 | 13 | COG6 | | | |  | | | |  | |  | |  | |  | |  | |  | |  | |  | |  | | |  | |  | |  | |
|  | |  | | |  | | |  | | | |  | |  | |  | |  | |  | |  | |  | |  | |  |  | |  | |  | |  |
|  | rs10213692 | 5 | ANKRD55 | | | |  | | |  | | | | | | | | | | | | | | | | | | | | | | | | | |
|  | rs27293 | 5 | | ERAP2 | | LNPEP | | |  | | | | | | | | | | | | | | | | | | | | | | | | | | |
|  | rs2847293 | 18 | | PTPN2 | |  | | |  | | | | | | | | | | | | | | | | | | | | | | | | | | |
|  | rs34536443 | 19 | | TYK2 | |  | | |  | | | | | | | | | | | | | | | | | | | | | | | | | | |
|  | rs7137828 | 12 | | SH2B3 | | ATXN2 | | |  | | | | | | | | | | | | | | | | | | | | | | | | | | |
|  | rs71624119 | 5 | | ANKRD55 | |  | | |  | | | | | | | | | | | | | | | | | | | | | | | | | | |
|  | rs72698115 | 1 | | ATP8B2 | | IL6R | | |  | | | | | | | | | | | | | | | | | | | | | | | | | | |
|  | rs7909519 | 22 | | IL2RA | |  | | |  | | | | | | | | | | | | | | | | | | | | | | | | | | |
|  | rs10194635 | 2 | | AFF3 | | LONRF2 | | |  | | | | | | | | | | | | | | | | | | | | | | | | | | |
|  | rs10280937 | 7 | | JAZF1 | |  | | |  | | | | | | | | | | | | | | | | | | | | | | | | | | |
|  | rs10849448 | 12 | | LTBR | |  | | |  | | | | | | | | | | | | | | | | | | | | | | | | | | |
|  | rs11074967 | 16 | | PRM1 | | RMI2 | | |  | | | | | | | | | | | | | | | | | | | | | | | | | | |
|  | rs11714843 | 3 | | TIMMDC1 | | CD80 | | |  | | | | | | | | | | | | | | | | | | | | | | | | | | |
|  | rs34132030 | 13 | | 13q14 | |  | | |  | | | | | | | | | | | | | | | | | | | | | | | | | | |
|  | rs66718203 | 16 | | PRM1 | | RMI2 | | |  | | | | | | | | | | | | | | | | | | | | | | | | | | |
|  | rs73300638 | 7 | | JAZF1 | |  | | |  | | | | | | | | | | | | | | | | | | | | | | | | | | |
|  | rs79893749 | 3 | | CCR1 | | CCR3 | | |  | | | | | | | | | | | | | | | | | | | | | | | | | | |
|  | rs9532434 | 13 | | COG6 | |  | | |  | | | | | | | | | | | | | | | | | | | | | | | | | | |

**Supplementary Table 1B**

| ABHD12 | C12orf30 | CLIC4 | FAS | IL17F | MAPK9 | PTPN2 | SLC38A2 | TSPAN9 |
| --- | --- | --- | --- | --- | --- | --- | --- | --- |
| ACAA2 | C12orf51 | CLLU1 | FBXL2 | IL2 | MAPKAPK5 | PTPN22 | SLC39A2 | TTC28 |
| ACAD10 | C5orf56 | CLLU1OS | FBXO25 | IL21 | MCM3 | PTPRS | SLC9A9 | TUBD1 |
| ACTA2 | C14orf181 | CLTC | FKHL18 | IL2RA | MCM5 | PTRH2 | SNORD58A | TYK2 |
| ADAD1 | C16orf46 | CNOT6 | FLJ10357 | IL2RB | MDK | PVT1 | SNORD58B | UBE2L3 |
| ADARB2 | C16orf61 | COG6 | FLJ14213 | IL6 | METT11D1 | PYGB | SNORD58C | UBP1 |
| AFF3 | C18orf32 | COL1A2 | FLJ39743 | IL6R | MGLL | RAB17 | SNORD67 | UNC13B |
| AHI1 | C20orf191 | COMMD9 | FLJ40434 | IRF1 | MIF | RAD51B | SNORD8 | VANGL1 |
| ALDH18A1 | C20orf94 | COX4I2 | FLJ78302 | JAG1 | MKRN2 | RAD51L1 | SORBS1 | VPS13B |
| ALDH1L1 | C22orf31 | CREB3L1 | FLT4 | JAZF1 | MOXD1 | RAF1 | SP8 | VPS8 |
| ALDH2 | C3orf1 | CTGF | FRG1B | JDP2 | MRGPRX4 | RASGEF1C | SPRED2 | WISP3 |
| AMBRA1 | C5 | CYP7B1 | FUT6 | JMJD1C | MYH13 | REEP3 | SRRM1 | XBP1 |
| ANGPT1 | C6orf10 | DCLRE1B | GAD2 | KCNK3 | MYLK2 | RGNEF | STAT1 | ZBP1 |
| ANKRD55 | C8orf42 | DEFB115 | GALNTL4 | KIAA0241 | MYO3A | RICH2 | STAT4 | ZBTB10 |
| ANTXR2 | C9orf93 | DEFB116 | GARNL4 | KIAA0652 | MYO5B | RMI2 | STK3 | ZFP36L1 |
| AP4B1 | CACNG3 | DEFB119 | GCSH | KIAA1109 | NANP | RNASE13 | STXBP6 | ZFP36L2 |
| AQP10 | CAND2 | DEFB121 | GFPT2 | KSR1 | NCK2 | RNASE2 | SUMO1P1 | ZNF160 |
| ARHGAP1 | CASQ2 | DEFB122 | GINS1 | KTELC1 | NDRG2 | RNASE7 | SUPT16H | ZNF217 |
| ARID2 | CC2D2B | DEFB123 | GLIS1 | LAMA1 | NFKBIZ | RNASE8 | SYT13 | ZNF219 |
| ATG5 | CCDC116 | DEFB124 | GLS | LAMB4 | NGF | RNFT1 | TAP2 | ZNF337 |
| ATMIN | CCDC117 | DGKZ | GPR98 | LGALS9 | NOS2A | RP4-691N24.1 | TCTN3 | ZNF365 |
| ATP6V1B1 | CCR1 | DHX40 | GRIK3 | LGI1 | NPTX2 | RPGRIP1 | TESK1 | ZNF408 |
| ATP8B2 | CCR3 | DKFZP434B0335 | hCG_1984468 | LIPG | NRBF2 | RPL17 | THADA | ZNF415 |
| ATXN2 | CCR5 | DMRTB1 | HIC2 | LNPEP | OR5AU1 | RPL6 | THUMPD2 | ZNF516 |
| AZIN1 | CD72 | DPP10 | HLA-DOB | LOC150223 | OSR2 | RPS6KB1 | TIGD1 | ZNF525 |
| BAIAP2L1 | CD80 | DTWD2 | HLA-DQA1 | LOC158381 | PDE6C | RSBN1 | TIMMDC1 | ZNF704 |
| BCAS1 | CDGAP | DUSP15 | HLA-DQA2 | LOC285359 | PEBP4 | RUNX1 | TMEM178 | ZNRF3 |
| BCAT1 | CDYL2 | DYM | HLA-DQB1 | LOC401463 | PHTF1 | RUNX3 | TMEM39A | ZPLD1 |
| BCL11A | CENPN | EDAR | HLA-DQB2 | LOC441208 | PI4KAP2 | RUSC2 | TMEM40 |  |
| BCL2L1 | CEP97 | EIF4E2 | HLA-DRA | LOC730112 | PITPNB | SCARNA17 | TMEM49 |  |
| BCL2L15 | CHD8 | ELAC2 | HLA-DRB1 | LOC91664 | PKD1L2 | SCGB3A1 | TMEPAI |  |
| BHLHB5 | CHD9 | ELTD1 | HLA-DRB5 | LONRF2 | PLA2G4A | SCML4 | TNFAIP3 |  |
| BRAP | CHEK2 | ENPP1 | HLA-DRB6 | LRP4 | PMP22 | SCNN1A | TP53I11 |  |
| BRI3 | CHRD | ENTPD1 | HM13 | LRRFIP1 | POLR3G | SFRS2IP | TPPP2 |  |
| BTG1 | CHRM4 | EPHA5 | HNRNPC | LSM5 | PRDM1 | SH2B3 | TPX2 |  |
| BTNL2 | CHRND | EPHB3 | HS3ST3A1 | LTBR | PRDM11 | SIT1 | TRAF1 |  |
| C10orf109 | CHRNG | ERAP2 | HSCB | LTF | PRKCQ | SLC11A1 | TRAFD1 |  |
| C10orf4 | CHST10 | ERP29 | ID1 | LYSMD3 | PRM1 | SLC22A4 | TSEN2 |  |
| C10orf59 | CKAP5 | F2 | IGSF5 | MAGEF1 | PSIMCT-1 | SLC22A5 | TSPAN18 |  |
| C11orf77 | CLASP2 | FAM55C | IL15 | MAGI3 | PTPN11 | SLC38A1 | TSPAN2 |  |
