# Supplementary material for: Network analysis identifies protein clusters of functional importance in juvenile idiopathic arthritis
Source: Arthritis Res Ther. 2014 May 8;16(3):R109. doi: 10.1186/ar4559 (PMC4062926; doi:10.1186/ar4559)
Supplement: Additional file 3: Table S3 — Network topology of the juvenile idiopathic arthritis (JIA) interactome: (A) Genes from the minimal essential network (MEN), that is, the top 10% of genes from the JIA interactome (ranked by degree (Deg) and bottleneck score (BN)). (B) JIA seed genes from the JIA interactome ranked by significance of deviation of observed network connectivity from expected (Disease Association Protein-Protein Link Evaluator (Dapple) algorithm, P ≤0.05). Orange = also present in minimal essential network (Figure 2B). [file ar4559-S3.docx]

| **Supplementary Table 3A** | | | | | | |  |  |  |  |  |
| --- | --- | --- | --- | --- | --- | --- | --- | --- | --- | --- | --- |
| Minimal Essential Network | | |  |  |  |  |  |  |  |  |  |
| **Symbol** | **BN** | **Deg** | **Symbol** | **BN** | **Deg** | **Symbol** | **BN** | **Deg** | **Symbol** | **BN** | **Deg** |
| UBC | 1889 | 171 | PTRH2 | 11 | 15 | SLC38A2 | 4 | 7 | CCT5 | 1 | 6 |
| NOS2 | 90 | 147 | THADA | 10 | 15 | HLA-DQA1 | 3 | 8 | RUVBL2 | 2 | 4 |
| STAT1 | 91 | 118 | CUL1 | 15 | 12 | RMI2 | 5 | 6 | STUB1 | 1 | 6 |
| FBXO25 | 81 | 117 | AMBRA1 | 9 | 17 | SH2B3 | 3 | 8 | RARA | 9 | 3 |
| UBE2L3 | 122 | 92 | TAP2 | 12 | 13 | RUSC2 | 6 | 6 | TRAF6 | 1 | 6 |
| RPL23 | 74 | 121 | CEP97 | 10 | 14 | ZNRF3 | 5 | 6 | TFAP4 | 6 | 4 |
| HNRNPC | 61 | 121 | CKAP5 | 8 | 17 | SPRED2 | 10 | 5 | IKBKG | 1 | 6 |
| PTPN11 | 76 | 92 | UNC13B | 12 | 12 | CAND1 | 1 | 15 | TP53BP1 | 1 | 6 |
| SRRM1 | 60 | 119 | F2 | 10 | 13 | PMEPA1 | 9 | 5 | FOS | 2 | 4 |
| RAF1 | 66 | 99 | PRDM1 | 9 | 15 | JMJD1C | 3 | 7 | COMMD9 | 5 | 4 |
| S100A9 | 76 | 74 | SCNN1A | 7 | 20 | RGNEF | 5 | 6 | TERF2 | 1 | 6 |
| MCM3 | 73 | 83 | GLS | 11 | 12 | CTGF | 3 | 7 | CCR1 | 2 | 4 |
| ATP6V1B1 | 69 | 78 | ZFP36L1 | 10 | 12 | CDK2 | 1 | 12 | METTL17 | 1 | 6 |
| CLTC | 54 | 101 | C20orf94 | 9 | 14 | DCLRE1B | 4 | 6 | CBL | 1 | 6 |
| RPL6 | 37 | 141 | KIAA0101 | 9 | 14 | SIRT7 | 1 | 12 | UBQLN4 | 8 | 3 |
| BCL2L1 | 56 | 70 | ZNF217 | 13 | 10 | RAD51B | 5 | 5 | LIG4 | 3 | 4 |
| SUPT16H | 49 | 68 | YWHAE | 8 | 15 | KHDRBS1 | 2 | 8 | ERBB2 | 1 | 5 |
| HSP90AA1 | 187 | 25 | TSEN2 | 10 | 11 | CUL5 | 2 | 8 | MAP3K5 | 1 | 5 |
| TNFAIP3 | 46 | 61 | MAPKAPK5 | 8 | 14 | NAA25 | 3 | 6 | CASP8 | 1 | 5 |
| ACTA2 | 52 | 49 | CENPN | 10 | 11 | GINS1 | 4 | 5 | MAGOH | 1 | 5 |
| FAS | 49 | 55 | PTPN2 | 9 | 12 | YWHAB | 3 | 6 | SMARCA4 | 1 | 5 |
| STK24 | 40 | 62 | MAGI3 | 10 | 11 | TP53 | 1 | 10 | NOTCH1 | 1 | 5 |
| MAPK9 | 46 | 53 | ATG13 | 8 | 13 | ZBTB10 | 3 | 6 | TRIM28 | 1 | 5 |
| TRAF1 | 34 | 56 | CLASP2 | 8 | 13 | HDAC1 | 2 | 7 | CUL4A | 1 | 5 |
| RUNX1 | 28 | 62 | DMRTB1 | 9 | 11 | VCAM1 | 1 | 10 | HNRNPA1 | 1 | 5 |
| CHEK2 | 29 | 55 | TESK1 | 8 | 12 | FBXL2 | 3 | 6 | JAZF1 | 4 | 4 |
| NINL | 39 | 44 | ALDH2 | 6 | 15 | CUL2 | 1 | 9 | SH3GL2 | 2 | 4 |
| RPL17 | 20 | 91 | CNOT6 | 8 | 12 | JUN | 5 | 5 | AKT1 | 1 | 5 |
| NCK2 | 34 | 39 | MDK | 8 | 12 | STAT4 | 4 | 5 | UBL4A | 1 | 5 |
| RPS6KB1 | 27 | 47 | LTBR | 9 | 11 | AP4B1 | 3 | 5 |  |  |  |
| STK3 | 28 | 39 | IL2RB | 4 | 17 | NEDD4 | 1 | 9 |  |  |  |
| SUMO2 | 41 | 24 | FN1 | 7 | 12 | SIT1 | 2 | 6 |  |  |  |
| ATXN2 | 27 | 31 | HLA-DRB1 | 5 | 15 | CSNK2A1 | 1 | 8 |  |  |  |
| ELAVL1 | 23 | 32 | LAMA1 | 9 | 9 | CTNNB1 | 3 | 5 |  |  |  |
| TYK2 | 21 | 29 | NRBF2 | 8 | 11 | ATP1B1 | 3 | 5 |  |  |  |
| S100A8 | 20 | 34 | PTPN22 | 7 | 11 | ITGA4 | 1 | 8 |  |  |  |
| MCM5 | 16 | 48 | BCAT1 | 9 | 9 | EGFR | 1 | 8 |  |  |  |
| BCL11A | 28 | 19 | UBP1 | 8 | 10 | SH3KBP1 | 1 | 8 |  |  |  |
| CHD8 | 21 | 23 | HM13 | 8 | 9 | FBXO6 | 3 | 5 |  |  |  |
| ACAA2 | 27 | 20 | HLA-DRB5 | 8 | 9 | VHL | 1 | 8 |  |  |  |
| SORBS1 | 18 | 24 | ZBP1 | 6 | 10 | FYN | 1 | 8 |  |  |  |
| XBP1 | 20 | 20 | HSP90AB1 | 5 | 11 | EP300 | 1 | 8 |  |  |  |
| ALDH18A1 | 24 | 18 | TECPR1 | 7 | 9 | PSMC5 | 5 | 4 |  |  |  |
| IRF1 | 16 | 25 | ZFPM1 | 6 | 9 | NGF | 3 | 5 |  |  |  |
| FRA10AC1 | 17 | 22 | CAND2 | 10 | 7 | RAD21 | 1 | 8 |  |  |  |
| APP | 14 | 25 | COG6 | 8 | 8 | SRC | 1 | 8 |  |  |  |
| EIF4E2 | 26 | 17 | JAG1 | 6 | 9 | XBP1P1 | 4 | 4 |  |  |  |
| PRKCQ | 13 | 25 | EPHB3 | 5 | 10 | PLA2G4A | 2 | 5 |  |  |  |
| BTG1 | 20 | 18 | MYLK2 | 6 | 9 | MYC | 1 | 7 |  |  |  |
| RUNX3 | 13 | 24 | ARRB1 | 7 | 8 | LCK | 1 | 7 |  |  |  |
| CCR5 | 18 | 17 | LTF | 6 | 9 | LRP4 | 3 | 5 |  |  |  |
| TPX2 | 13 | 23 | CUL3 | 2 | 18 | SMURF1 | 1 | 7 |  |  |  |
| DGKZ | 16 | 18 | CCR3 | 9 | 7 | BRCA1 | 1 | 7 |  |  |  |
| LRRFIP1 | 17 | 17 | AZIN1 | 6 | 8 | NEDD8 | 1 | 7 |  |  |  |
| ID1 | 14 | 18 | VCP | 4 | 10 | RAC1 | 3 | 5 |  |  |  |
| ELAC2 | 12 | 22 | KSR1 | 5 | 9 | CD72 | 2 | 5 |  |  |  |
| ESR1 | 17 | 16 | CHRD | 7 | 8 | HDAC5 | 1 | 7 |  |  |  |
| ZNF408 | 13 | 19 | BAIAP2L1 | 3 | 11 | GRIK3 | 4 | 4 |  |  |  |
| SUMO1 | 17 | 16 | YWHAQ | 3 | 11 | MDM2 | 1 | 7 |  |  |  |
| PTPRS | 12 | 20 | FLT4 | 6 | 8 | YWHAZ | 1 | 7 |  |  |  |
| HLA-DRA | 20 | 15 | UBD | 9 | 6 | AMH | 4 | 4 |  |  |  |
| IGF2BP1 | 9 | 41 | ARHGAP1 | 3 | 11 | THUMPD2 | 3 | 4 |  |  |  |
| FASN | 9 | 40 | POLR3G | 7 | 7 | HGS | 1 | 6 |  |  |  |
| ARRB2 | 77 | 10 | HLA-DRB3 | 3 | 11 | ANXA11 | 4 | 4 |  |  |  |
| ATG5 | 16 | 15 | C5 | 7 | 7 | SIRT3 | 2 | 5 |  |  |  |
| ARID2 | 13 | 17 | STAT3 | 4 | 9 | MAPK6 | 1 | 6 |  |  |  |
| MIF | 11 | 18 | VANGL1 | 7 | 7 | TRAF3 | 1 | 6 |  |  |  |
| PYGB | 13 | 17 | MAGEF1 | 6 | 7 | HSPA8 | 1 | 6 |  |  |  |
| COPS5 | 16 | 14 | SCAF11 | 3 | 10 | EEF1A1 | 1 | 6 |  |  |  |
| LSM5 | 12 | 16 | GRB2 | 3 | 10 | CTBP2 | 1 | 6 |  |  |  |
| BRAP | 11 | 16 | LNPEP | 6 | 6 | TRAFD1 | 2 | 4 |  |  |  |
| JDP2 | 14 | 14 | ZNF219 | 4 | 8 | SHC1 | 1 | 6 |  |  |  |
| DHX40 | 14 | 13 | CLIC4 | 5 | 7 | EIF4A3 | 1 | 6 |  |  |  |

| **Supplementary Table 3B** | | | | | | |
| --- | --- | --- | --- | --- | --- | --- |
| **Gene** | **P-Value** |  |  |  |  |  |
| **IL2RB** | 0.0002 |  |  |  |  |  |
| **STAT1** | 0.0002 |  |  |  |  |  |
| **C12orf51** | 0.0002 |  |  |  |  |  |
| **ENTPD1** | 0.0002 |  |  |  |  |  |
| **DCLRE1B** | 0.0006 |  |  |  |  |  |
| **PTPN11** | 0.0008 |  |  |  |  |  |
| **TRAFD1** | 0.0008 |  |  |  |  |  |
| **SIT1** | 0.0010 |  |  |  |  |  |
| **IL15** | 0.0014 |  |  |  |  |  |
| **IL2** | 0.0014 |  |  |  |  |  |
| **ZNRF3** | 0.0016 |  |  |  |  |  |
| **IL21** | 0.0018 |  |  |  |  |  |
| **BAIAP2L1** | 0.0020 |  |  |  |  |  |
| **TYK2** | 0.0022 |  |  |  |  |  |
| **SH2B3** | 0.0036 |  |  |  |  |  |
| **IL6** | 0.0036 |  |  |  |  |  |
| **NFKBIZ** | 0.0036 |  |  |  |  |  |
| **ENPP1** | 0.0038 |  |  |  |  |  |
| **IL2RA** | 0.0042 |  |  |  |  |  |
| **RAF1** | 0.0046 |  |  |  |  |  |
| **CDGAP** | 0.0048 |  |  |  |  |  |
| **PTPN22** | 0.0052 |  |  |  |  |  |
| **VPS13B** | 0.0052 |  |  |  |  |  |
| **SLC39A2** | 0.0058 |  |  |  |  |  |
| **TMEM39A** | 0.0074 |  |  |  |  |  |
| **KSR1** | 0.0088 |  |  |  |  |  |
| **MYH13** | 0.0088 |  |  |  |  |  |
| **IL6R** | 0.0110 |  |  |  |  |  |
| **PTPN2** | 0.0112 |  |  |  |  |  |
| **MOXD1** | 0.0124 |  |  |  |  |  |
| **RNFT1** | 0.0136 |  |  |  |  |  |
| **NDRG2** | 0.0145 |  |  |  |  |  |
| **HLA-DQB1** | 0.0149 |  |  |  |  |  |
| **AHI1** | 0.0151 |  |  |  |  |  |
| **HLA-DRB5** | 0.0153 |  |  |  |  |  |
| **HLA-DQA2** | 0.0155 |  |  |  |  |  |
| **HLA-DOB** | 0.0223 |  |  |  |  |  |
| **HLA-DQA1** | 0.0235 |  |  |  |  |  |
| **CACNG3** | 0.0248 |  |  |  |  |  |
| **HLA-DRA** | 0.0266 |  |  |  |  |  |
| **CHRNG** | 0.0323 |  |  |  |  |  |
| **CLIC4** | 0.0335 |  |  |  |  |  |
| **TNFAIP3** | 0.0345 |  |  |  |  |  |
| **PEBP4** | 0.0355 |  |  |  |  |  |
| **RASGEF1C** | 0.0365 |  |  |  |  |  |
| **MIF** | 0.0370 |  |  |  |  |  |
| **LTF** | 0.0384 |  |  |  |  |  |
| **LRRFIP1** | 0.0398 |  |  |  |  |  |
| **PLA2G4A** | 0.0398 |  |  |  |  |  |
| **ATMIN** | 0.0404 |  |  |  |  |  |
| **CEP97** | 0.0406 |  |  |  |  |  |
| **GINS1** | 0.0455 |  |  |  |  |  |
| **STAT4** | 0.0480 |  |  |  |  |  |
| **KCNK3** | 0.0500 |  |  |  |  |  |
