# Supplementary material for: Network analysis identifies protein clusters of functional importance in juvenile idiopathic arthritis
Source: Arthritis Res Ther. 2014 May 8;16(3):R109. doi: 10.1186/ar4559 (PMC4062926; doi:10.1186/ar4559)
Supplement: Additional file 5: Table S5 — Oligoarticular versus control gene expression: overlap of genes in oligoarticular juvenile idiopathic arthritis (JIA) compared to controls present in both gene expression datasets analysed. Datasets described in Additional file 2: Table S2. Red = up-regulated in JIA, green = down-regulated in JIA. [file ar4559-S5.docx]

| **Supplementary Table 2** | | | | | | | | |
| --- | --- | --- | --- | --- | --- | --- | --- | --- |
| **Accession** | **Study** | **Sample Numbers** | **Control Numbers** | **Comparisons** | **Number of Probe-sets Associated with Comparison** | **Genes** | **Confounding Factors** | **Chip Type** |
| **GDS711** | **Juvenile rheumatoid arthritis expression profiles in mononuclear cells** | Oligo PBMC = 5 | Control PBMC = 11 | PBMC Oligo v Control | 1737 | 1568 | x | U95Av2 |
|  |  | Poly PBMC = 15 | “ | PBMC Poly v Control | 4304 | 3593 | x |  |
|  |  | Oligo SFM = 5 | “ | PBMC Oligo v Poly | 645 | 581 | x |  |
|  |  | Poly SFM = 15 | “ | SFM Oligo v Poly | 674 | 602 | x |  |
| **GSE11083** | **Childhood Onset Rheumatic Disease Gene Expression Profile** | Poly PBMC = 13 | PBMC control = 15 | PBMC Poly v Control | 6897 | 4607 | Gender | Affymetrix Human Genome U133 Plus 2.0 Array |
|  |  | Poly Neut = 13 | Neut Control = 13 | Neut Poly v Control | 4387 | 2735 | Gender |  |
| **GSE20307** | **Biological Similarities Exist between Oligoarticular and Polyarticular Subtypes of JIA Based on Age at Onset** | PBMC RF-Poly = 44 | PBMC Control = 56 | PBMC Poly v Control | 5202 | 3415 | Ethnicity, Gender, Age | Affymetrix Human Genome U133 Plus 2.0 Array |
|  |  | PBMC Oligo = 40 | “ | PBMC Oligo v Control | 5799 | 3670 | Ethnicity, Gender, Age |  |
|  |  | “ | PBMC RF-Poly = 44 | PBMC Oligo v Poly | 2835 | 2006 | Ethnicity, Gender, Age |  |
| **GSE17755** | **Human peripheral blood cells: autoimmune diseases vs. healthy individuals** | PBMC Poly = 6 | PBMC Control = 8 | PBMC Poly v Control | 6397 | 3180 | Gender, Age | Hitachisoft AceGene Human Oligo Chip 30K 1 Chip Version |
|  |  |  |  |  |  |  |  |  |
